# LSD1 contributes to programmed oocyte death by regulating the transcription of autophagy adaptor SQSTM1/p62

**DOI:** 10.1111/acel.13102

**Published:** 2020-02-19

**Authors:** Meina He, Tuo Zhang, Zijian Zhu, Shaogang Qin, Huarong Wang, Lihua Zhao, Xinran Zhang, Jiayi Hu, Jia Wen, Han Cai, Qiliang Xin, Qirui Guo, Lin Lin, Bo Zhou, Hua Zhang, Guoliang Xia, Chao Wang

**Affiliations:** ^1^ State Key Laboratory of Agrobiotechnology College of Biological Sciences China Agricultural University Beijing China; ^2^ Key Laboratory of Ministry of Education for Conservation and Utilization of Special Biological Resources in the Western China Ningxia University Yinchuan China

**Keywords:** autophagy, H3K4me2, LSD1, oocyte loss, p62, primordial follicle pool

## Abstract

In female mammals, the size of the initially established primordial follicle (PF) pool within the ovaries determines the reproductive lifespan of females. Interestingly, the establishment of the PF pool is accompanied by a remarkable programmed oocyte loss for unclear reasons. Although apoptosis and autophagy are involved in the process of oocyte loss, the underlying mechanisms require substantial study. Here, we identify a new role of lysine‐specific demethylase 1 (LSD1) in controlling the fate of oocytes in perinatal mice through regulating the level of autophagy. Our results show that the relatively higher level of LSD1 in fetal ovaries sharply reduces from 18.5 postcoitus (dpc). Meanwhile, the level of autophagy increases while oocytes are initiating programmed death. Specific disruption of LSD1 resulted in significantly increased autophagy and obviously decreased oocyte number compared with the control. Conversely, the oocyte number is remarkably increased by the overexpression of *Lsd1* in ovaries. We further demonstrated that LSD1 exerts its role by regulating the transcription of *p62* and affecting autophagy level through its H3K4me2 demethylase activity. Finally, in physiological conditions, a decrease in LSD1 level leads to an increased level of autophagy in the oocyte when a large number of oocytes are being lost. Collectively, LSD1 may be one of indispensible epigenetic molecules who protects oocytes against preterm death through repressing the autophagy level in a time‐specific manner. And epigenetic modulation contributes to programmed oocyte death by regulating autophagy in mice.

## Graphical abstract

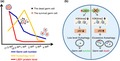

## 1 INTRODUCTION

In female mammals, only a restricted number of oocytes are reserved within the initially established pool of primordial follicles (PFs) (Oktem & Urman, 2010), which determines the reproductive lifespan of individuals (Oktem & Urman, 2010). The development of fetal ovaries as well as the number of live oocytes for the formation of PFs within newborn ovaries is under precise control (Wang, Zhou, & Xia, 2017). Inadequate follicles in the initially established pool are related to primary premature ovarian insufficiency (POI), which is diagnosed by amenorrhea before 40 years of age (Qin, Jiao, Simpson, & Chen, 2015). Approximately half to two‐thirds of oocytes are lost physiologically at middle‐late gestation (Tilly, 2001). In line with this finding, approximately 7 million oocytes degenerate to 1–2 million at week 20 of gestation in the human neonatal ovary (BAKER, 1963). The reasons for the programmed cell death (PCD) of oocytes could be varied and need substantial study. Furthermore, in‐depth studies of the causes of oocyte programmed loss will provide a better understanding of the pathogenic mechanism of POI or even female infertility (Rodrigues, Limback, Mcginnis, Plancha, & Albertini, 2009; Sun, Sun, Dyce, Shen, & Chen, 2017). To date, apoptosis and autophagy have been reported to be involved in the process of oocyte loss, although the underlying mechanisms for each type of cell death require substantial study (Alton & Taketo, 2007; Gawriluk et al., 2011; Tanner, Blute, Brachmann, & Mccall, 2011).

The transition from fetal to neonatal life is a critical period for the development of organ systems, including heart muscle, diaphragm, alveolar cells, skin (Kuma et al., 2004), thymus (Mizushima, Yamamoto, Matsui, Yoshimori, & Ohsumi, 2004), and ovaries (Gawriluk et al., 2011), because they have to adapt to starvation after birth and experience non‐apoptotic or apoptotic death (Edinger & Thompson, 2004; Norberg, Orrenius, & Zhivotovsky, 2010). Autophagy is a conserved mechanism that maintains cellular homeostasis through degrading and recycling long‐lived proteins, damaged cytoplasmic organelles (Mizushima & Levine, 2010). Autophagy is also active during the process of oocyte development, including follicular assembly and perinatal oocyte loss (Barth, Szabad, Hafen, & Kohler, 2011; Tu et al., 2019). For instance, autophagy‐related gene 7 (*Atg7*) is a key molecule that is pivotal for fetal mouse ovary development, and *Atg7* loss results in infertility in adults (Song et al., 2015). Similarly, *beclin 1* deficiency in 1‐day postpartum (dpp) mouse ovaries resulted in as much as 50%‐60% loss of oocytes (Gawriluk et al., 2011). Moreover, the autophagic substrate p62 is a multifunctional adaptor protein that regulates the packing and delivery of polyubiquitinated, misfolded, aggregated proteins, and dysfunctional organelles for their clearance in mammalian and *Drosophila* cells (Gawriluk et al., 2011). However, whether p62 is also actively involved in mouse oocyte PCD needs further study.

Recently, the importance of epigenetic modification in regulating somatic cell reprogramming as well as early embryo development has been widely reported (Liu et al., 2018; Matoba & Zhang, 2018; Yu et al., 2017). However, only a handful of studies have indicated that epigenetic modification plays an important role in controlling oocyte PCD during PF pool establishment (Sun et al., 2017, 2018). Most recent studies uncovered the potential role of LSD1, in mediating autophagy in various cell types (Ambrosio et al., 2017; Byun et al., 2017; Periz et al., 2015). LSD1 is the first identified lysine‐specific demethylase that specifically marks H3K4me1/2 and/or H3K9me1/2 via a FAD‐dependent oxidative reaction (Shi et al., 2004). LSD1 plays crucial roles in the germ lines of multiple organisms. In *Drosophila*, LSD1 deficiency leads to the complete absence of oocytes and severe defects in spermatogenesis (Lee & Spradling, 2014; Stefano, Ji, Moon, Herr, & Dyson, 2007). In *C. elegans*, disruption of the LSD1 ortholog spr‐5 leads to progressive sterility in both sexes over generations, resulting from failure to erase H3K4me2 in primordial oocytes (Katz, Edwards, Reinke, & Kelly, 2009). Additionally, in mammals, LSD1 is closely related to autophagy‐mediated cancer growth, liver cell development, and embryogenesis (Byun et al., 2017; Wang et al., 2007; Wang, Long, et al., 2017). However, whether LSD1 participates in the PCD of oocytes in perinatal mice and which signaling pathway(s) are activated remain uncertain.

Here, we presented evidence demonstrating that LSD1 controlled global H3K4me2 at a lower level in oocytes before the initiation of autophagy‐induced oocyte loss. The time‐specific decrease in LSD1 levels from the fetal to neonatal period in the ovaries correlates with the upregulated expression of p62. Compared with the control, LSD1 inhibition in the fetal ovary in vitro while the PF pool is established substantially compromises the number of oocytes, accompanied by a high level of autophagy. Therefore, a stepwise decrease in the levels of LSD1 may be indispensable for timely control of the degree of oocyte death induced by autophagy in prenatal mouse ovaries.

## 2 RESULTS

### 2.1 LSD1 is essential for the survival of oocytes around the time of birth in mice

**Figure 1:**
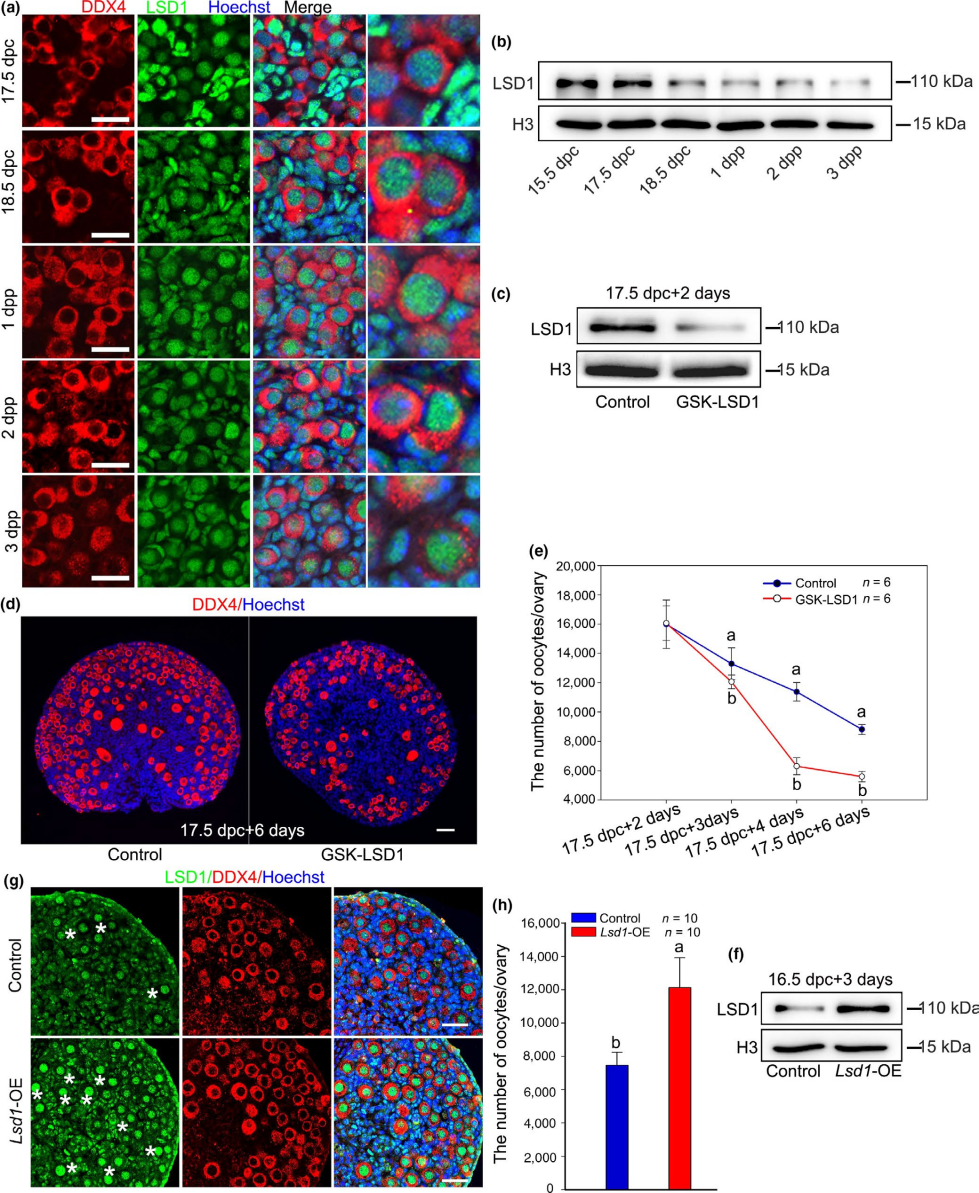
LSD1 is essential for the survival of oocytes around the time of birth in mice. (a) LSD1 was mainly localized to the nucleus of the oocytes and somatic cells. (b) The total protein level of LSD1 in mouse ovaries from 15.5 dpc to 3 dpp. (c) GSK‐LSD1 significantly suppressed the expression of LSD1 in cultured ovaries. (d) Supplementation with GSK‐LSD1 significantly reduced the oocytes number. (e) The time‐dependent inhibitory effects of GSK‐LSD1 on the oocytes number. (f) A demonstration of the transfection efficiency of *Lsd1*‐OE plasmid. (g, h) The positive effect of *Lsd1*‐OE on preventing oocyte loss and the surviving oocytes (asterisks). LSD1: green; the oocyte marker DDX4: red; nuclei: blue. The different letters above the bars indicate significant differences. Scale bars, 25 μm

To address the physiological significance of LSD1 during oocyte loss and PF formation, we first analyzed the cellular localization and expression pattern of LSD1 during PF formation. Immunofluorescence results showed that LSD1 was located in the nuclei of both somatic cells and oocytes in fetal and neonatal mouse ovaries (Figure 1a). Western blotting revealed that the expression level of LSD1 in the ovaries was decreased in a time‐specific manner from 15.5 dpc to 3 dpp. Notably, from 18.5 dpc, LSD1 decreased sharply to 3 dpp (Figure 1b). This dynamic expression pattern of LSD1 motivated us to study its potential roles in massive oocyte loss during PF formation.

To explore the role of LSD1 during the period of PF formation beginning at 17.5 dpc and finishing at 4 dpp, we utilized GSK‐LSD1, a LSD1‐specific inhibitor, to culture fetal ovaries in vitro with DMSO as a control. Western blot demonstrated that GSK‐LSD1 significantly inhibited LSD1 when 17.5 dpc ovaries were cultured for 2 days (Figure 1c). GSK‐LSD1‐treated ovaries contained significantly fewer PFs than did the control ovaries after 6 days culture (Figure 1d). To see the dynamic change of oocyte number after treated with GSK‐LSD1, 17.5 dpc ovaries were cultured for different days. Compared with the control, GSK‐LSD1 began having a remarkable influence on oocytes number from 2 days in culture (Figure 1e). Inhibition of LSD1 by SP2509, another LSD1 reversible inhibitor, leads to both the decrease of LSD1 protein level and oocytes loss in cultured ovaries (Figure S1). These findings obtained from applying SP2509 are consistent with those obtained from GSK‐LSD1.

Alternatively, to further clarify the role of LSD1 in controlling oocytes number, 16.5 dpc ovaries were transfected with the *Lsd1‐*overexpression (*Lsd1*‐OE) plasmid and a scrambled plasmid sequence as the control. The expression efficiency of the transfected plasmids was confirmed by Western blotting after cultured for 3 days (Figure 1f). After being cultured for 7 days, *Lsd1*‐OE resulted in significantly more available oocytes than did the control (Figure 1g,h). The effect of overexpression of *Lsd1* on oocyte number was opposite to that of inhibition of LSD1 by GSK‐LSD1.

Generally, if oogonia mitosis or oocyte meiosis progression is affected, oocytes will be abnormal or even dead. Some oocytes in fetal mouse ovaries initiate meiosis as early as 13.5 dpc. Therefore, to confirm whether the action of LSD1 on preventing premature oocyte death has temporal specificity and whether it is correlated with oogonia mitosis and oocyte meiosis progression, 13.5 dpc ovaries were cultured with GSK‐LSD1 until 17.5 dpc. GSK‐LSD1 had no obvious effects on the number of oocytes (Figure S2A,B). Moreover, oocytes expressing proliferating cell nuclear antigen (PCNA) were unaffected (Figure S2C,D). In addition, after ovaries were cultured from 15.5 dpc to 18.0 dpc or from 17.5 dpc to 1 dpp and oocyte meiosis progression was examined by a chromosome spread assay, meiosis progression was unaffected (Figure S2E,F). Therefore, LSD1 is not essential for oocyte mitosis and meiosis.

### 2.2 LSD1 acts as a H3K4me2 demethylase in regulating the fate of oocytes perinatally

**Figure 2:**
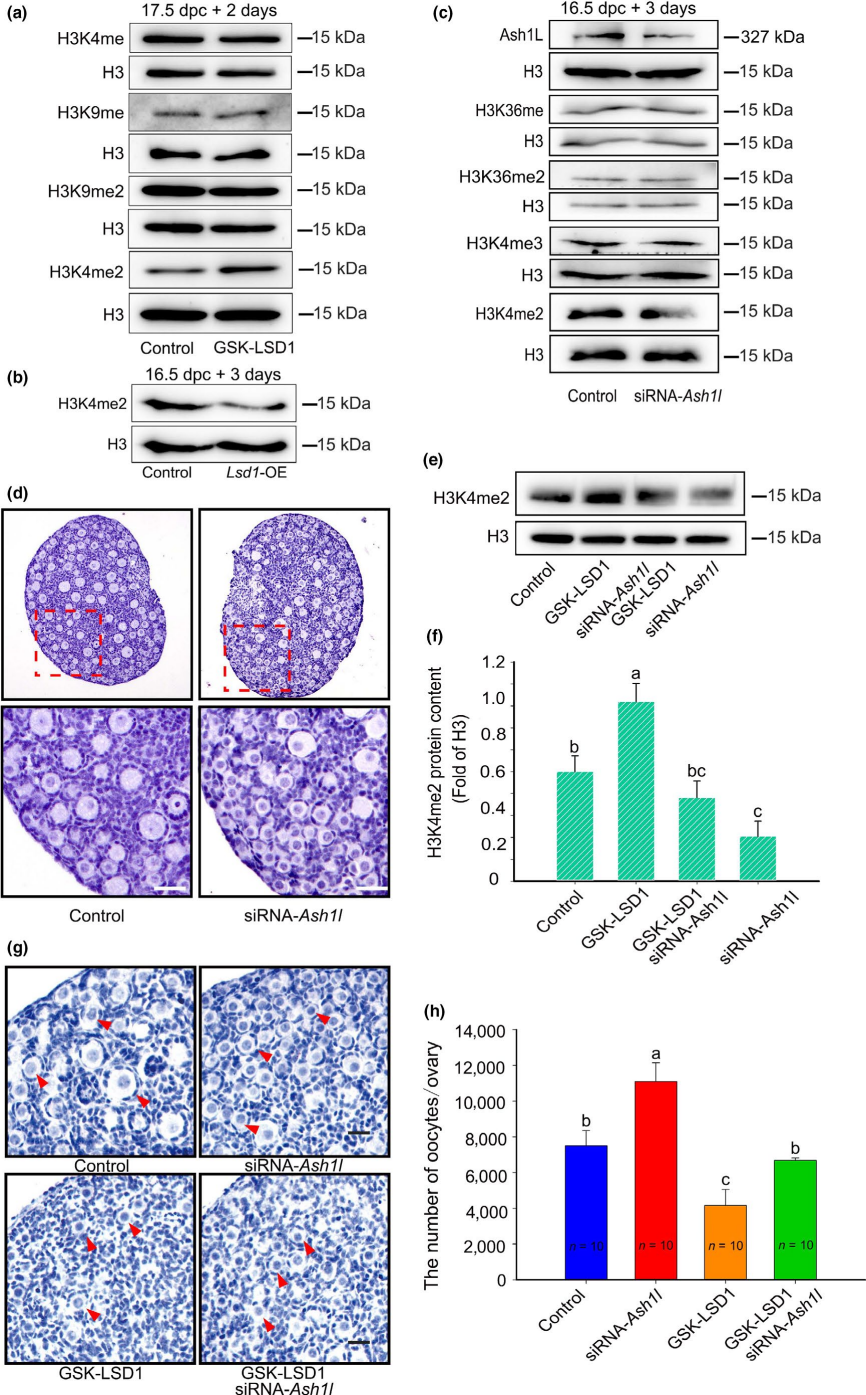
LSD1 acts as a H3K4me2 demethylase in regulating the fate of oocytes perinatally. (a) The inhibition of LSD1 significantly increased the level of H3K4me2, while the levels of H3K4me1 and H3K9me1/2 were unaffected. (b) The overexpression of *Lsd1* significantly increased the level of H3K4me2. (c) The validation of the efficiency of *Ash1l* knockdown on Ash1L level. The knockdown of *Ash1l* significantly decreased the level of H3K4me2, while the levels of H3K4me3 and H3K36me1/2 were unaffected. (d) Remarkably, more oocytes were observed after the silencing of *Ash1l* in 16.5 dpc ovaries cultured for 7 days. (e‐h) The underlying effect of GSK‐LSD1 on the promotion of oocyte survival after *Ash1l* was knocked down in 16.5 dpc ovaries. One day after ovaries were injected with siRNA‐*Ash1l* or scrambled siRNA, the medium was supplemented with GSK‐LSD1 or DMSO, and the cells were cultured for an additional 3 or 6 days. (e, f) The rescue of oocyte survival (arrows) by GSK‐LSD1 was demonstrated and analyzed. (g, h) The rescue of H3K4me2 levels by GSK‐LSD1 when *Ash1l* was knocked down*.* The different letters above the bars indicate significant differences. Scale bars, 25 μm

To determine which lysine binding sites function in the ovaries, we examined the change of H3K4me1/2 and H3K9me1/2 after ovaries were treated with GSK‐LSD1 for 2 days. Notably, in most LSD1 substrates, only H3K4me2 levels were significantly changed. The level of H3K4me2 in cultured ovaries was either upregulated by the inhibition of LSD1 (Figure 2a) or downregulated by the overexpression of *Lsd1* (Figure 2b), indicating that LSD1 likely functions in fetal ovaries via its H3K4me2 demethylase activity.

Theoretically, if the H3K4me2 level is critical for oocyte fate, histone H3K4me2 methyltransferases, such as ASH1 like histone lysine methyltransferase (Ash1L) may also actively participate in the process. Indeed, compared with the control, knockdown of *Ash1l* resulted in decreased levels of H3K4me2. However, after knockdown of *Ash1l*, other lysine sites, namely H3K36me, H3K36me2 and H3K4me3 did not change significantly (Figure 2c). Moreover, after knockdown of *Ash1l*, the number of oocytes significantly increased compared with the control group (Figure 2d,f). Furthermore, to explore whether LSD1 and Ash1L have reciprocal inhibitory effects in controlling oocyte number, we performed a combined assay including control, GSK‐LSD1, *Ash1l* siRNA, and GSK‐LSD1 plus *Ash1l* siRNA. Silencing *Ash1l* significantly restored the effect of GSK‐LSD1 on oocytes number (Figure 2e–H). Thus, the level of H3K4me2 balanced by methyltransferases and demethyltransferases was vital for directing the fate of oocytes.

### 2.3 Apoptosis is involved in massive oocytes death but is not the major cause

To clarify whether apoptosis was involved in the oocyte loss, we performed the following assays. Since apoptosis‐inducing factor (AIF) is an effector in the caspase‐independent pathway of apoptosis, the level of AIF was examined first. Western blotting and immunofluorescence results showed that the expression level and location of AIF were unaffected by GSK‐LSD1 supplementation (Figure S3A,B). In contrast, the level of active caspase‐3, which was located in the nucleus of oocytes, was obviously higher in GSK‐LSD1 supplementation than in the control (Figure S3C,D). We have also applied the TUNEL assay following LSD1 inhibition accordingly. The results showed that the number of TUNEL‐positive cells was increased after the inhibition of LSD1 (Figure S3E). Then, to identify whether apoptosis is important for oocytes death, we added Z‐VAD‐fmk, a pan‐caspase inhibitor, to the media to culture 17.5 dpc ovaries. Western blotting results showed that GSK‐LSD1 alone significantly elevated the level of active caspase‐3, but this effect could be inhibited by Z‐VAD‐fmk (Figure S3F,G). During simultaneous inhibition of LSD1 and apoptosis, the number of oocytes was increased but not significantly (Figure S3H,I). Therefore, apoptosis is involved in massive oocyte death after LSD1 inhibition but may not be the major cause.

### 2.4 Inhibition of LSD1 results in extensive autophagy in oocytes and is related to oocytes death

**Figure 3:**
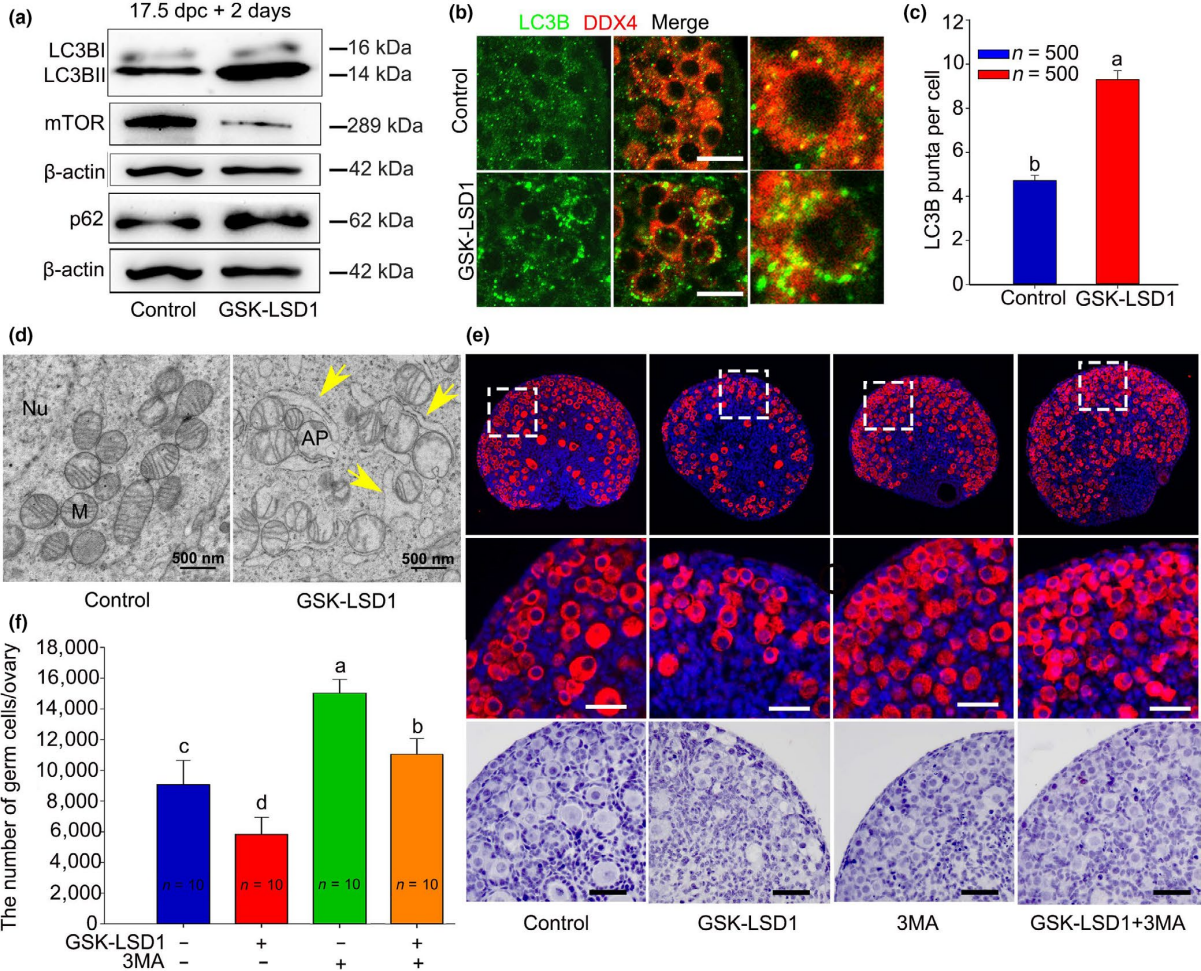
Inhibition of LSD1 results in extensive autophagy in oocytes and is related to oocytes death. (a) The autophagy‐related signaling pathway was changed. (b, c) LC3 puncta aggregation was observed in the cytoplasm of oocytes 2 days after GSK‐LSD1 treatment. LC3B: green; oocytes: red; nuclei: blue. (d) Autophagosomes were observed under TEM analysis, 2 days after GSK‐LSD1 treatment (arrows). AP, autophagosome; M, mitochondria; Nu, nucleus. (e, f) The rescue of oocytes number by 3MA in GSK‐LSD1‐treated ovaries. (e) Morphological evidence. (f) Statistical evidence. The different letters above the bars indicate significant differences. Scale bars, 25 μm

To further ascertain whether autophagy was involved in oocytes death, we observed changes in LC3B in response to LSD1 levels. LC3B serves as an autophagy indicator by conversion of LC3BI into LC3BII. After 17.5 dpc ovaries were cultured with GSK‐LSD1 for 2 days, dramatically increased levels of LC3B conversion and decreased levels of mTOR were observed (Figure 3a). Similar results were obtained by immunofluorescence staining where many LC3 puncta were aggregated in the cytoplasm of GSK‐LSD1‐treated oocytes (Figure 3b,c). Furthermore, transmission electron microscopy (TEM) analysis revealed autophagosomes or autolysosomes containing degraded organelles, which were primarily mitochondria‐like organelles (Figure 3d).

To provide more evidence demonstrating that autophagy is pivotal for oocyte loss perinatally, we used an autophagy inhibitor, 3MA. Based on an assay to determine the optimized concentration of 3MA (Figure S4), 2.5 mM 3MA was added into the culture media with GSK‐LSD1. After 17.5 dpc ovaries were cultured for 6 days, the number of oocytes that survived in the 3MA group was remarkably higher than that in the control group. Importantly, during simultaneous inhibition of LSD1 and autophagy, the number of oocytes was significantly increased compared with that during GSK‐LSD1 treatment (Figure 3e,f). In conclusion, excessive autophagy caused by LSD1 deficiency leads to autophagic cell death of oocytes.

### 2.5 LSD1 inhibition does not block autophagic flux

**Figure 4:**
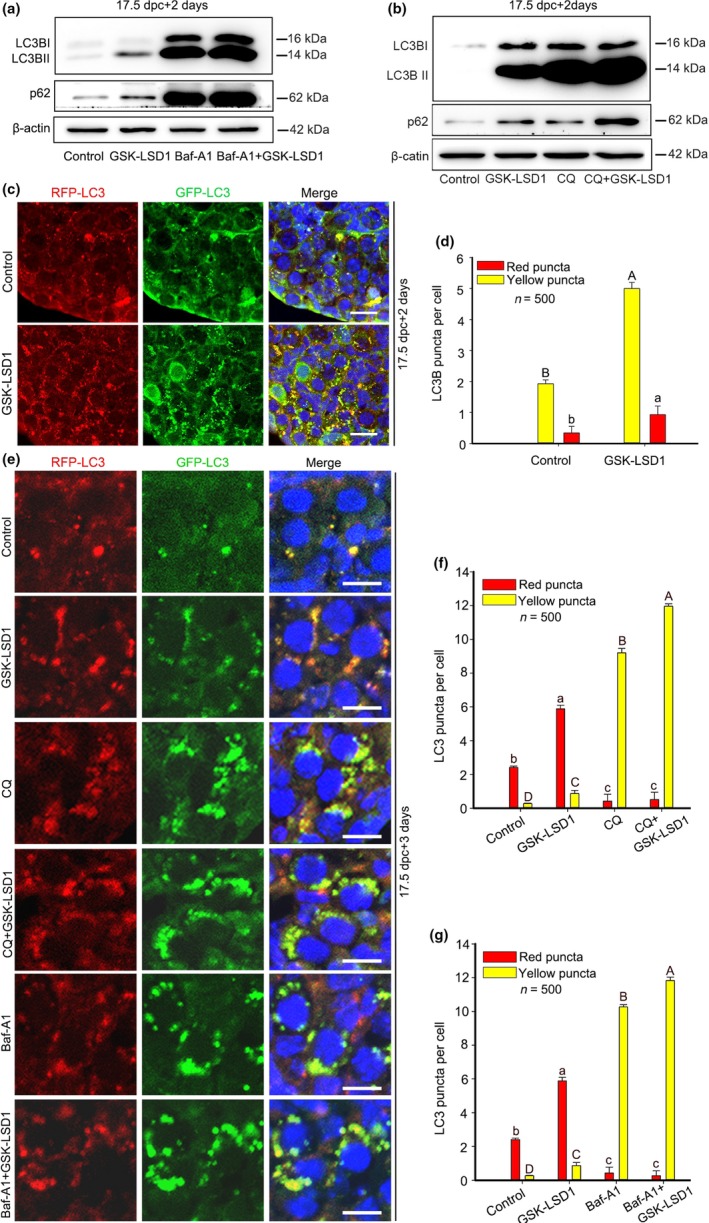
LSD1 inhibition does not block autophagic flux. (a, b) The levels of LC3BII and p62 increased remarkably in ovaries that were treated with CQ/Baf‐A1 compared with the respective controls. Additionally, if ovaries were simultaneously treated with GSK‐LSD1 and CQ/Baf‐A1 cultured for 2 days, the protein levels of LC3II and p62 increased to even higher levels. (c, d) LC3 puncta aggregation was observed in the cytoplasm of oocytes from RFP‐EGFP‐LC3 mice 2 days after GSK‐LSD1 treatment in RFP‐EGFP‐LC3 mice. Combined GFP and RFP fluorescence represents phagophores and autophagosomes, while the puncta emitting only an RFP signal represent autolysosomes. Nuclei: blue. The different letters above the bars indicate significant differences. (e) LC3 yellow puncta aggregation was observed in the cytoplasm of oocytes after 3 days of treatment with CQ/Baf‐A1 plus GSK‐LSD1. (f, g) The counting results of LC3 puncta. GFP‐LC3: green; RFP‐LC3: red; nuclei: blue. The different letters above the bars indicate significant differences. Scale bars, 10 μm

The level of endogenous p62 protein is turned by autophagic degradation. However, we found that when LSD1 was inhibited, the level of p62 was increased (Figure 3a). To find out whether the accumulation of p62 is caused by autophagy induction or by blocked autophagic degradation, we assessed the autophagic flux. Fetal mouse ovaries from 17.5 dpc were treated with chloroquine diphosphate (CQ) or bafilomycin A1 (Baf‐A1), two lysosome inhibitors capable of blocking autophagic degradation. The Western blot results showed that the levels of LC3BII and p62 were both elevated after CQ or Baf‐A1 supplementation. Treatment with CQ plus GSK‐LSD1 or Baf‐A1 plus GSK‐LSD1 contributed to even higher levels of the two proteins in cultured ovaries compared with that induced by GSK‐LSD1 treatment alone (Figure 4a,b).

To further examine the effect of LSD1 inhibition on autophagic flux, we employed RFP‐EGFP‐LC3 mice to investigate the fusion of autophagosomes with lysosomes to form autolysosomes. This sensor consists of an acid‐sensitive GFP mutant, which fluoresces at an acidic pH, and an acid‐insensitive RFP, which fluoresces at both acidic and neutral pHs, permitting the progression from an autophagosome with a neutral pH to an autolysosome with an acidic pH to be monitored. Consistent with previous results, we found that both GFP signals combined with RFP signals and RFP fluorescence signals alone were greater in GSK‐LSD1 ovaries than in control ovaries after two days of culture, indicating the accumulation of autophagosomes in GSK‐LSD1 ovaries (Figure 4c,d). After another 24‐hr treatment, the majority of puncta remaining in the ovaries of the control and the GSK‐LSD1 groups were red puncta. The majority of puncta in the CQ‐ and Baf‐A1‐treated groups as well as in the CQ plus GSK‐LSD1‐treated and the Baf‐A1 plus GSK‐LSD1‐treated groups were yellow puncta (Figure 4e). Treatment with CQ plus GSK‐LSD1 or Baf‐A1 plus GSK‐LSD1 contributed to even more puncta in cultured ovaries compared with that induced by GSK‐LSD1 treatment alone (Figure 4f,g). It can be concluded that GSK‐LSD1 stimulates autophagosome formation but does not block autophagic flux in cultured mouse ovaries.

### 2.6 LSD1 inhibition does not block the degradation function of lysosomal nor affect the activity of ubiquitin protease system

To detect observable changes within oocytes in the aspects of lysosomes morphology, the number of lysosomes and the fusion of lysosomes and autophagosomes, LysoTracker red was used to stain lysosomes and autolysosomes. The results showed that the size of the lysosomes did not change, but the number of lysosomes increased obviously in the ovaries in which LSD1 was inhibited. And the autophagosomes and lysosomes can fuse together after LSD1 was inhibited (Figure S5A,B). Considering that the capacity of lysosomal degradation is a rate‐limiting factor for autophagic flux, we next assessed the function of lysosomes. LysoSensor™ Green was used to measure the pH of lysosomes. The results showed that the LysoSensor dye showed stronger fluorescence in ovaries in which LSD1 was inhibited than in control ovaries (Figure S5C). For further detecting the acidification of lysosomes, the results showed that the activity of lysosomal acid phosphatase 2 (ACP2) increased in ovaries in which LSD1 was inhibited (Figure S5D). Moreover, we examined the activity of cathepsin B (CTSB), CTSB is a lysosomal cysteine protease that was increased after LSD1 inhibition in ovaries (Figure S5E). Concomitant with the increase in CTSB, cathepsin L (CTSL) was also increased (Figure S5F). Taken together, these results reveal that LSD1 inhibition does not block the degradation capability of autolysosomes and lysosomes.

To further verify the regulatory relationships between LSD1, ubiquitin‐proteasome system (UPS) activity and p62, we performed several experiments. Briefly, the ovaries were treated with either proteasome inhibitor MG132 or protein synthesis inhibitor cycloheximide (CHX), respectively. We found that after ovaries were treated with MG132 plus GSK‐LSD1, the level of p62 protein was significantly higher than those ovaries treated with either MG132, or GSK‐LSD1 alone (Figure S6A). In addition, the level of p62 protein in CHX plus GSK‐LSD1 group was significantly lower than those ovaries treated with either control or GSK‐LSD1 (Figure S6B). Taken together, the raised protein level of p62 in response to LSD1 inhibition may not be caused by the decreased activity of UPS.

### 2.7 Elevated H3K4me2 promotes *p62* transcription and causes oocyte death

To determine which genes that participate in autophagy are involved in oocyte loss in response to LSD1 changes, we systematically analyzed the transcription levels of *Ulk1*, *p62,* and *ATG*s. The mRNA levels of *p62* and *Atg3* were notably changed (Figure S7A), but only the protein level of p62 was significantly elevated in response to GSK‐LSD1 (Figure 3a and Figure S7B). When LSD1 was inhibited or *Lsd1* was overexpressed, the level of p62 was increased (Figure 3a and Figure S7D) or decreased accordingly (Figure S7C,E).

**Figure 5:**
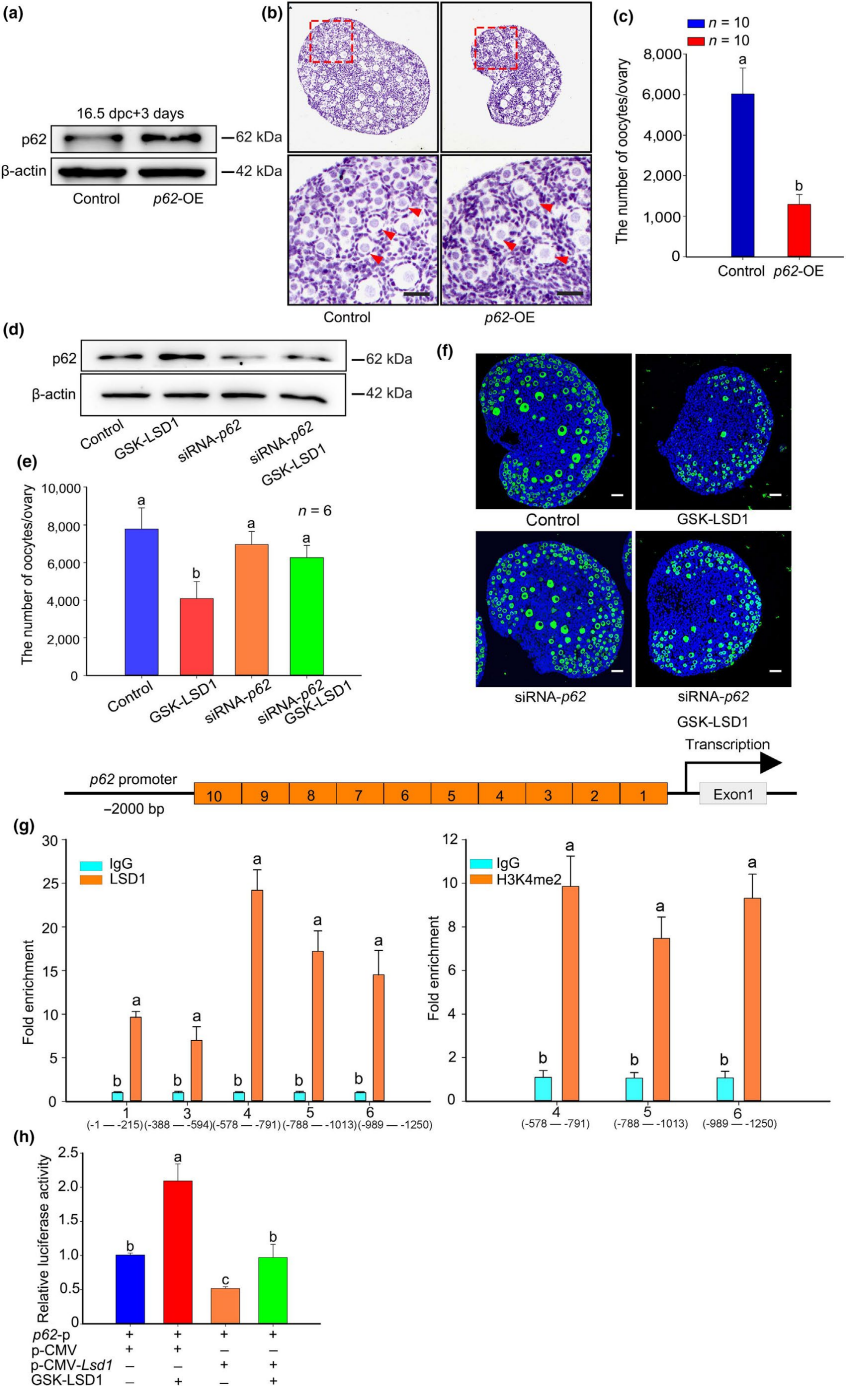
Elevated H3K4me2 promotes *p62* transcription and causes oocyte death. (a) The overexpression of *p62* significantly increased the level of p62. (b) The ovaries in the *p62*‐OE group exhibited fewer oocytes (arrows) than that exhibited by the control. (c) Statistical evidence. (d–f) The underlying effect of GSK‐LSD1 on the promotion of oocyte survival after *p62* was knocked down in 16.5 dpc ovaries. One day after ovaries were injected with siRNA‐*p62* or scrambled siRNA, the medium was supplemented with GSK‐LSD1 or DMSO, and the ovaries were cultured for an additional 3 or 6 days. (g) ChIP‐qPCR analysis showed that both LSD1 and H3K4me2 occupied the *p62* promoter. (h) Double luciferase reporter gene assay showed LSD1 regulates the transcription activity of p62 in vitro. The different letters above the bars indicate significant differences. Scale bars, 25 μm

To clarify whether the increased level of p62 induced by LSD1 inhibition causes oocyte death, 16.5 dpc ovaries were transfected with a *p62*‐OE plasmid. The expression efficiency of the transfected plasmids was confirmed by Western blotting after cultured for 3 days (Figure 5a). The number of oocytes was remarkably decreased (Figure 5b,c), as predicted. Western blotting showed that the LC3B conversion was similar to the changes that occurred when LSD1 was inhibited (Figure S7F).

To explore the potential role of p62 in regulating autophagy and the fate of germ cells, 16.5 dpc ovaries were transfected with a siRNA‐*p62* plasmid. The expression efficiency of the transfected plasmids was confirmed by Western blotting after cultured for 3 days (Figure 5d). The results showed that when GSK‐LSD1 was added to the culture of *p62* knockdown ovaries, the protein levels of p62 were increased compared with those of the untreated *p62* knockdown ovaries (Figure 5d). The phenotype of the cultured ovaries treated with siRNA‐*p62* alone or siRNA‐*p62* plus GSK‐LSD1 was assessed by immunofluorescence. The results showed that the decreased number of germ cells induced by GSK‐LSD1 treatment was elevated when p62 was knocked down simultaneously. However, the number of germ cells was unchanged in the *p62* knockdown ovaries compared with the control; the reason for this change requires further study (Figure 5e,f). According to these results, it is speculated that p62 may be one of the downstream molecules of LSD1, but not the only one.

To clarify how LSD1 regulates p62, we studied the cellular location of p62 under physiological conditions. The results showed that p62 was located in the cytoplasm of oocytes and somatic cells (Figure S7G,H). The localization was unaffected by either LSD1 inhibition or *Lsd1* overexpression (Figure S7D,E). To address how LSD1 modulates p62 responsiveness, we performed ChIP‐qPCR analysis. Based on the results, LSD1 can directly bind to the region of −1250 bp to −1 bp region of the *p62* promoter (Figure 5g). In this study, we found that LSD1 regulates autophagy in ovary through its H3K4me2 demethylase activity, we wondered whether the level of p62 is regulated by both LSD1 and H3K4me2. Thus, we performed ChIP‐qPCR analysis, too. Based on the results, H3K4me2 can directly bind to the region of −1250 bp to −578 bp region of the *p62* promoter (Figure 5g). Moreover, we digested whole ovaries and then isolated oocytes and somatic cells to perform ChIP‐qPCR. The results of the efficiency test for oocytes and somatic cells isolation are shown in Figure S8A,B. The ChIP‐qPCR results showed that both LSD1 and H3K4me2 can bind to the −1250 bp to −578 bp region and the −215 bp to −1 bp region of the p62 promoter in oocytes (Figure S8C,D). In somatic cells, both LSD1 and H3K4me2 can bind to the region −1250 bp to region −388 bp of the *p62* promoter (Figure S8E,F). Furthermore, we performed double luciferase reporter gene assay and investigated whether LSD1 regulates the transcription activity of *p62* in vitro. It showed that the overexpression of *Lsd1* could significantly decrease the transcription activity of *p62*. Importantly, this decreased activity could be reversed by the inhibitor of LSD1 (Figure 5h). These results suggested that the elevated H3K4me2 in response to LSD1 inhibition may activate the transcription of *p62*. In turn, p62 largely accumulates in the cytoplasm and contributes to oocyte loss.

### 2.8 Autophagy is involved in massive oocyte loss during the establishment of the PF pool in vivo

**Figure 6:**
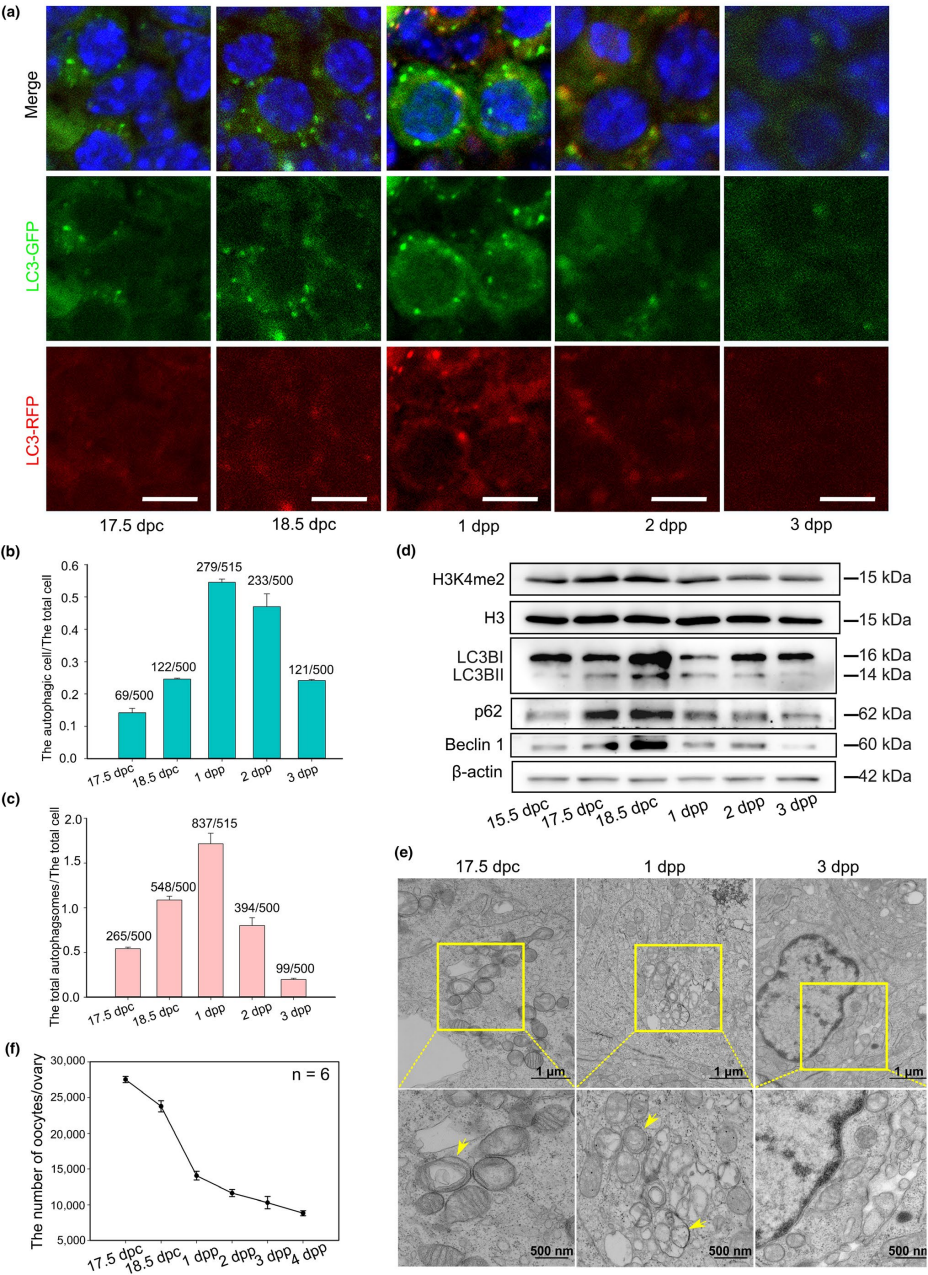
Autophagy is involved in massive oocyte loss during the establishment of the PF pool in vivo. (a) Morphological analysis of autophagic activity in vivo. Ovaries were obtained at 17.5 dpc, 18.5 dpc, 1 dpp, 2 dpp, and 3 dpp from RFP‐EGFP‐LC3 mice. Nuclei: blue. Scale bars, 10 μm. (b, c) The quantification of autophagic cells and autophagosomes. (d) The expression patterns of LC3B, H3K4me2, Beclin 1, and p62 in vivo. (e) Autophagosomes were observed by TEM, and ovaries were obtained 17.5 dpc, 1 dpp, and 3 dpp. (f) The counting result of the oocytes at different time points in vivo. Scale bars, 10 μm

**Figure 7:**
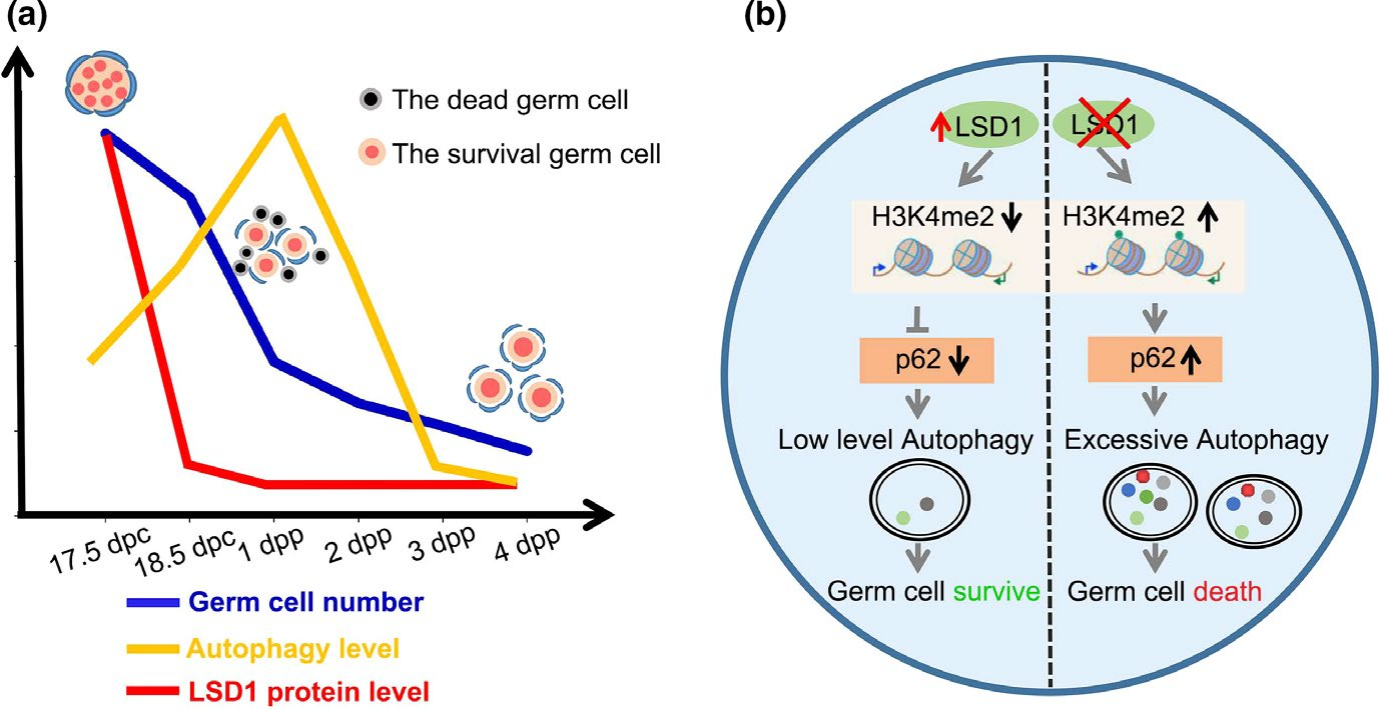
A proposed model depicting how LSD1 regulates autophagy to protect oocytes from loss during primordial follicle formation. (a) A summary of the relationship between LSD1, autophagy, and the number of oocytes during primordial follicle formation. (b) A diagram showing the function and regulation mechanism of LSD1 during primordial follicle formation. LSD1 regulates the transcription of autophagy adaptor and substate p62 by its H3K4me2 demethylase activity

To verify the important role of autophagy in oocyte loss during the establishment of the PF pool, we examined the autophagy level under physiological conditions in a RFP‐EGFP‐LC3 mouse model. Briefly, from 17.5 dpc to 3 dpp ovaries were collected. The formation of GFP‐LC3 puncta was first upregulated from 17.5 dpc to 1 dpp (Figure 6a) and then gradually decreased to basal levels from 2 dpp to 3 dpp. Accordingly, the levels of autophagy were higher at 18.5 dpc to 2 dpp than at 3 dpp (Figure 6b,c). In addition, the amount of membrane‐bound LC3II transiently increased at 18.5 dpc and returned to basal levels at 3 dpp, while Beclin 1 and p62 increased at 15.5 dpc, peaked at 18.5 dpc, and decreased at 3 dpp (Figure 6d). Importantly, more autophagosomes or autolysosomes containing degraded organelles like mitochondria were observed through TEM in 1 dpp ovaries than in 3 dpp ovaries (Figure 6e). Coincidently, Western blotting results also showed that the H3K4me2 level increased starting at 15.5 dpc, peaked at 18.5 dpc, and then decreased at 3 dpp (Figure 6d). In addition, ChIP‐qPCR analysis proved that p62 was regulated by LSD1 and H3K4me2 (Figure 5g). Finally, oocyte loss was accelerated soon after H3K4me2 was elevated and slowed down after H3K4me2 was decreased (Figure 6f). In summary, LSD1 exerts its role by regulating the transcription of *p62* and affecting autophagy level through its H3K4me2 demethylase activity. Thus, autophagy may be indispensable for oocyte PCD during PF formation in mice (Figure 7).

## 3 DISCUSSION

In most mammals, programmed loss of oocytes occurs briefly around the time of birth (Byun et al., 2017; Wang, et al., 2007; Wang, et al., 2017), but the reasons and the mechanisms remain uncertain. Several processes take effort to the oocyte loss and different PCDs may have various effects on oocyte PCD under specific situations before the pool of PFs is established. Cellular apoptosis is the first recognized process responsible for oocyte PCD (Alton & Taketo, 2007; Coucouvanis, Sherwood, Carswellcrumpton, Spack, & Jones, 1993; Felici et al., 1999; Kuma et al., 2004). Alternatively, autophagy actively participates in the sharp decrease in oocytes number (Barth et al., 2011; Lobascio, Klinger, Scaldaferri, Farini, & Felici, 2007; Rodrigues et al., 2009). Here, we have proved that both apoptosis and autophagy are involved in oocyte PCD and that autophagy‐induced oocyte death is repressed in a time‐specific manner by the epigenetic enzyme LSD1 in mice.

LSD1 as an epigenetic regulator of the autophagic pathway is pivotal in protecting oocytes from massive loss. Studies including ours have proved that LSD1 localizes to the nucleus of cells (Kahl et al., 2006; Lim et al., 2010) and is critical for the negative regulation of genes essential for the autophagic process by recruiting to the promoters of genes like *Atg3*, *Atg10*, transcription factor EB (*Tfeb)*, sestrin 2 (*Sesn2*), and *p62* (Ambrosio, Ballabio, & Majello, 2019; Ambrosio & Majello, 2018; Ambrosio et al., 2017; Byun et al., 2017; Periz et al., 2015; Sardiello et al., 2009; Settembre et al., 2011; Wang, Long, et al., 2017). LSD1 importing to the nucleus is related to its N‐terminal nuclear localization signal motifs (Jin et al., 2014). Interestingly, the underlying mechanisms of LSD1‐induced autophagy vary considerably depending on specific circumstances (Ambrosio & Majello, 2018; Byun et al., 2017; Wang, Long, et al., 2017). In neuroblastoma cells, LSD1 represses SESN2 expression, which hampers mTORC1 activity (Ambrosio et al., 2017). In liver cells, LSD1 mediates fed‐state hormone signaling in preventing autophagy (Byun et al., 2017). Although SESN2 expression in fetal ovaries was increased after LSD1 inhibition, the overexpression of *Sesn2* did not affect oocytes number (data not shown). Alternatively, based on our results, p62 may be the key target of LSD1 during this process.

Autophagy is implicated in many systems, such as heart, liver, and pancreas (Mizushima et al., 2004). p62 involved in autophagy by virtue of its different functional domains (Kirkin et al., 2009). On one hand, p62 enhances autophagy activity by disrupting the interactions between Bcl‐2 and Beclin 1 (Zhou et al., 2013). On the other hand, the interaction between p62 and LC3B enables the cargo‐p62 complex to be selectively tethered to the autophagosome (Tung, Hsu, Lee, Huang, & Liao, 2010). Here, the changed levels of H3K4me2, p62, and LC3 are synergistic with changes of LSD1 in vivo when the stepwise loss of oocytes is initiated. Inhibition of LSD1 resulted in significant premature oocyte loss and remarkably higher level of p62. In agreement with our findings, inhibition of LSD1 in gynecologic malignancies increases p62 levels and activates autophagy (Tung et al., 2010). However, there are some differences in the mechanism. According to Chao's and Lan's studies, LSD1 destabilizes target protein independent of its demethylase activity (Chao et al., 2017; Lan et al., 2019). In our study, LSD1 regulates the transcription of *p62* depending on its demethylase activity. This suggests that the mechanism of LSD1's regulation on downstream molecules is very complex under different conditions. Since the degradation of p62 is a common marker for autophagic processes (Dikic, 2017), the protein level of p62 is generally regulated by both transcription and degradation. We have demonstrated here that autophagic degradation and ubiquitin‐proteasome system (UPS) are not significantly affected by LSD1 inhibition and that the increased p62 resulted from transcription activation. Collectively, our results imply that the fine‐tuned LSD1‐controlled p62 expression may be a new autophagy‐induced oocyte PCD pathway.

The identity of the type of cell death for oocyte loss around the time of birth is controversial. Previously, apoptosis is considered the major pattern for oocyte PCD. That is not only because low‐level apoptosis occurs before birth, but because increase or decrease follicle numbers occurred in postnatal mice bearing systemic deletions of pro‐apoptotic or anti‐apoptotic genes (Matikainen et al., 2002; Perez et al., 1999). However, not all apoptosis‐related gene deletions, such as *Bax*, resulted in significant oocyte PCD alterations after birth, alternatively apoptosis eliminates oocytes with genetic or meiotic pairing errors (Tilly, 2001). This indicates that there exist other mechanisms responsible for extensive oocyte degeneration and follicles formation (Greenfeld, Pepling, Babus, Furth, & Flaws, 2007; Perez et al., 1999). Our study together with others have demonstrated that autophagy‐induced PCD of oocytes may be pivotal for oocyte loss and functional follicles formation in mice without affecting meiosis because the number of oocytes recovered after inhibiting autophagy was significantly higher than that of apoptosis (Ignazio et al., 2018; Rodrigues et al., 2009; Tu et al., 2019). Apoptosis appears to eliminate oocytes with genetic or meiotic pairing errors, while autophagy helps oocytes to form functional follicles.

In summary, although the initiation and the end of oocyte PCD within fetal ovaries remain to be elucidated, this study suggests that LSD1 negatively regulated autophagy through the p62 pathway may be indispensable for programmed oocyte loss around birth in mice. The findings contribute to a better understanding of the physiological mechanism of oocyte PCD and may help to find the causative factors impacting POI in humans.

## 4 EXPERIMENTAL PROCEDURES

### 4.1 Animals

All CD1 mice and C57B6/J mice were purchased from the Laboratory Animal Center of the Institute of Genetics and Developmental Biology, Beijing, China. Female mice were mated with males overnight and checked for a vaginal plug the following morning. The presence of a vaginal plug was considered 0.5 day postcoitus (dpc). The day after partum was considered to be 1 day postpartum (dpp). All animal experiments conformed to the guidelines and regulatory standards of the Institutional Animal Care and Use Committee of China Agricultural University.

The CAG‐RFP‐EGFP‐LC3 transgenic mice (Stock No: 027139) was purchased from the Jackson Laboratory. Genotyping primers used are listed in Table S1.

### 4.2 Ovary isolation and culture

Ovaries were separated by microdissection from the mesonephros or ovarian capsule in prechilled PBS (10 mM, pH 7.4) under a stereomicroscope (ZSA302, COIC). The isolated ovaries were cultured in 6‐well culture dishes (NEST Biotechnology) with basic DMEM/F‐12 medium (Gibco, Life Technologies) and Penicillin‐Streptomycin at 37°C in a 5% CO_2_, 95% air atmosphere with saturated humidity.

Ovaries were cultured in either medium with dimethylsulfoxide (DMSO) or medium supplemented with inhibitor. The concentration of inhibitors used in this study: GSK‐LSD1‐2Hcl (S7574; Selleck): 64 μM; 3MA (S2767; Selleck, USA): 2.5 mM; Z‐VAD‐fmk (S7023; Selleck): 50 μM; Chloroquine diphosphate (C6688; Sigma): 10 μM; Bafilomycin A1 (S1413; Selleck): 60 nM; MG132 (S2619; Selleck): 5 μM; Cycloheximide (HY‐12320; MedChemExpress): 2 nM; and SP2509 (S7680; Selleck): 5 nM.

### 4.3 Plasmid construction

To overexpress the *Lsd1* and *p62* gene, *Lsd1* and *p62* were cloned into pCMV‐Blank (D2602; Beyotime). The promoter of *p62* was cloned into pGL3‐Basic (E1751; Promega). Golden star t6 super PCR mix was purchased from Beijing Tsingke Co., Ltd. All of the constructs were verified by sequencing. Primers are listed in Table S1.

### 4.4 RNA interference (RNAi) and gene overexpression

To assure that siRNAs and overexpression vectors would be transfected into the inner cells of fetal ovaries, 0.5 μl of the siRNAs or overexpression vectors were injected into isolated 16.5 dpc ovaries using glass pipettes with a stereomicroscope. After the ovaries were full of liquid, electrotransfection (ECM2001; BTX, CA) was performed by applying three 5‐ms‐long quasi‐square pulses at a pulse‐field strength of up to 30 V/cm. The *Ash1l* knockdown siRNA and the *p62* knockdown siRNA were purchased from Thermo Scientific Inc. Ovaries were cultured for 72 hr to test the transfection efficiency of protein levels, or for 7 days for histological examination and oocyte counting. Primers used are listed in Table S1.

### 4.5 Histological sections and oocyte counts

Ovaries were fixed in cold 4% paraformaldehyde (PFA) overnight, embedded in paraffin, and serially sectioned at 5 μm. The sections were stained with hematoxylin, and the number of oocytes was counted in every fifth section; to estimate the total numbers of oocytes in each ovary, the sum was multiplied by five.

### 4.6 Immunofluorescence

Ovaries were fixed in 4% PFA overnight, embedded in paraffin, and sectioned at 5 μm. After dewaxing, rehydration, and high‐temperature (92°C) antigen retrieval with 0.01% sodium citrate buffer (pH 6.0), the sections were blocked with 10% normal serum for 60 min at room temperature and immunostained with primary antibodies overnight at 4°C. Subsequently, after rinsing thoroughly with PBS, the slides were then incubated with Alexa Fluor 488‐ or 555‐conjugated secondary antibodies (1:100; Invitrogen) and Hoechst 33342 (1:1000; B2261, Sigma) at 37°C for 1 hr. Slides were then rinsed in PBS and sealed in antifade fluorescence mounting medium (Applygen) with coverslips. Sections were examined and photographed using a Nikon Eclipse 80i digital fluorescence microscope or a Nikon A1 laser scanning confocal microscope. Primary antibodies and dilutions used are presented in Table S2.

### 4.7 Western blotting

Proteins were separated on a 10% SDS‐PAGE gel and then transferred onto polyvinylidene fluoride membranes (IPVH00010; Millipore). SDS‐PAGE and immunoblots were performed following standard procedures using a Mini‐PROTEAN Tetra Cell System (Bio‐Rad). The antibodies used are listed in Table S2.

### 4.8 Lysosomal acid phosphatase assay

Lysosomal acid phosphatase 2 (ACP2) activity was assayed by a commercially available kit (P0326, Beyotime) according to the manufacturer's instructions. The absorbance was measured at 405 nm by microplate reader (SPARK, TECAN).

### 4.9 Lysosome labeling with LysoTracker red DND‐99

Lysosomes were labeled with LysoTracker Red DND‐99 (L7528; Life Technologies) according to the manufacturer's protocol. Briefly, LysoTracker Red was diluted 1:13,300 from the stock solution (1 mM in anhydrous DMSO) and was added to cultured cells, and the cells were incubated at 37°C for 12 hr. Images were taken using a Nikon A1 laser scanning confocal microscope.

### 4.10 Lysosome labeling with LysoSensor Green DND‐189

Lysosomes were labeled with LysoSensor Green DND‐189 (L7535; Life Technologies) according to the manufacturer's protocol. Briefly, LysoSensor Green was diluted 1:1,000 from the stock solution (1 mM in anhydrous DMSO) and was added to cultured cells, and the cells were incubated at 37°C for 12 hr. Images were taken using a Nikon A1 laser scanning confocal microscope.

### 4.11 CTSB/CSTL activity assay

CTSB/CSTL was measured using the Magic Red Cathepsin B/L detection kit (937/941, ImmunoChemistry Technologies, USA). Control ovaries and ovaries in which LSD1 was inhibited were cultured in 6‐well plates with different treatment times, as indicated. Then, the ovaries were collected into 0.6‐ml tubes and incubated with 50 μl of 0.25% trypsin at 37℃ for 5 min. The ovaries were pipetted up and down to digest them into single‐cell suspensions. A total of 2.5 μl of fetal bovine serum was added to tubes to terminate the digestion reaction. The single‐cell suspensions were centrifuged at 800 *g* for 5 min. The supernatant was discarded, and the cells were resuspended in 620 μl of DMEM‐F12. Then, the cells were loaded with Magic Red Cathepsin B/L reagent at 37°C for 1 hr. To quantify CTSB/CTSL activity, the fluorescence of the Magic Red Cathepsin B/L probe was measured using a fluorescence microplate reader (SPARK, TECAN) according to the manufacturer's manual.

### 4.12 Real‐Time RT‐PCR

Total RNA was isolated from mouse ovaries with TRIzol (Invitrogen, Life Technologies). One microgram of total RNA was used to synthesize cDNA according to manufacturer's instructions (Promega Reverse Transcription System, Promega). qRT‐PCR was performed using a Power SYBR Green PCR Master Mix (Applied Biosystems, Life Technologies) with ABI 7500 Real‐Time PCR system (Applied Biosystems). Primers are listed in Table S1.

### 4.13 Chromatin Immunoprecipitation (ChIP)

ChIP assays were performed using a MAGNA ChIP kit (17‐371RF; Millipore) according to the manufacturer's protocol. Immunoprecipitations were performed with cross‐linked chromatin from 18.5 dpc mouse ovaries and either anti‐H3K4me2 antibody, anti‐LSD1 antibody, or normal IgG. The enriched DNA was quantified by real‐time PCR. Primers used are listed in Table S1.

### 4.14 Isolation of somatic cells and oocytes

Mouse fetal ovaries were collected in 1.5‐ml tubes at 18.5 dpc and incubated with 400 μl of 0.25% trypsin at 37°C for 10–15 min. The ovaries were pipetted up and down for 3 min to digest them into single‐cell suspensions. A total of 50 μl of fetal bovine serum was added to each tube to terminate the digestion reaction. The single‐cell suspensions were centrifuged at 3,000 rpm for 5 min. The supernatant was discarded, and the ovarian cells were washed with 1 ml of PBS. The cells were centrifuged once again, and the supernatant was discarded. The ovarian cells were resuspended in 2 ml of DMEM/F12 with 5% FBS and supplemented with a 1% modified insulin‐transferrin‐selenium solution (ITS, 51500056; Life Technologies). The ovarian cells were transferred to 6‐well plates and cultured at 37°C and 5% CO_2_ for 5–6 hr. The plates were gently shaken, and loosely adhered oocytes were released. The supernatant was collected and centrifuged to collect the oocytes. The ovarian cells that had adhered to the culture plate were collected and centrifuged to collect somatic cells.

### 4.15 Low cell number chromatin immunoprecipitation

The collected oocytes and somatic cells were performed ChIP assays with Low Cell# ChIP Kit ^TM^ Protein G (C01010073; Diagenode) according to the manufacturer's protocol.

### 4.16 Double luciferase assays

Human embryonic cells 293FT were cultured in Dulbecco's modified Eagle's medium with 10% fetal bovine serum supplemented with 100 IU/ml penicillin and 100 IU/ml streptomycin. 293FT cells were transfected with the *Lsd1* overexpression vector or the pCMV‐blank control vector, *p62* luciferase reporter vectors, and pTK‐Ranilla vector (E2241; Promega) at a ratio of 10:4:1 using the Lipofectamine 3000 transfection reagent (L3000015; Invitrogen, Life Technologies) according to the manufacturer's protocol. 293FT cells were cultured 24 hr after transfection. Luciferase activity was measured using a Dual‐Luc Assay Kit (T002 Vigorous) with fluorescence microplate reader (SPARK, TECAN).

### 4.17 Transmission Electron Microscopy (TEM)

Ovaries were fixed in 2.5% glutaraldehyde (within 0.2 M PBS, pH = 7.2) overnight at 4°C. Then, the treated ovaries were processed and wrapped in epoxypropane resin following standard TEM procedures.

### 4.18 Chromosome spreads

SYCP3 antibody was used to identify the chromosomal axial elements at meiosis prophase I. The stages of meiotic prophase I were evaluated based on the appearance of axial elements according to previous studies (Prieto et al., 2004). In total, 300 oocytes from three ovaries were counted on each slide and repeated for three animals. The primary antibodies are presented in Table S2.

### 4.19 Statistical analysis

All experiments were repeated at least three times, and the values are presented as the means ± *SEM*. The data were analyzed by *t* test or ANOVA and considered statistically significant at *p* < .05.

## CONFLICT OF INTEREST

The authors declare no competing interests.

## AUTHOR CONTRIBUTIONS

Meina He, Tuo Zhang, Guoliang Xia, and Chao Wang designed the research. Meina He, Tuo Zhang, Zijian Zhu, Shaogang Qin, Xinran Zhang, Jiayi Hu, Jia Wen, Han Cai, Qirui Guo, Lin Lin, and Bo Zhou performed the research. Meina He, Tuo Zhang, Chao Wang, Huarong Wang, Lihua Zhao, and Hua Zhang analyzed the data. Meina He, Tuo Zhang, and Chao Wang wrote the paper. All authors have seen and approved the final version.

## Data Availability

Data sharing is not applicable to this article as no new data were created or analyzed in this study.
