# Supplementary figures and images for: LSD1 contributes to programmed oocyte death by regulating the transcription of autophagy adaptor SQSTM1/p62
Source: Aging Cell. 2020 Feb 19;19(3):e13102. doi: 10.1111/acel.13102 (PMC7059144; doi:10.1111/acel.13102)

**A**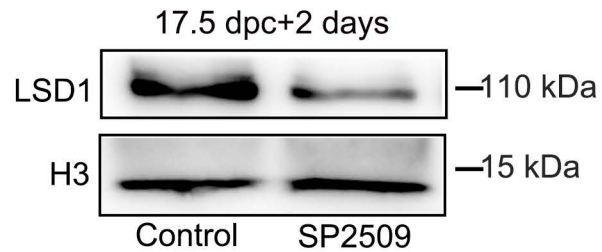**C**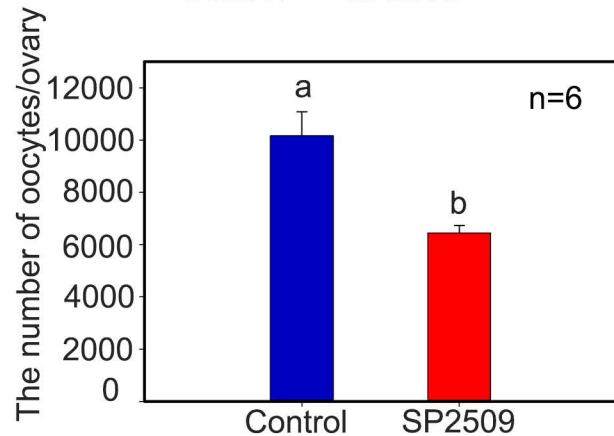**B**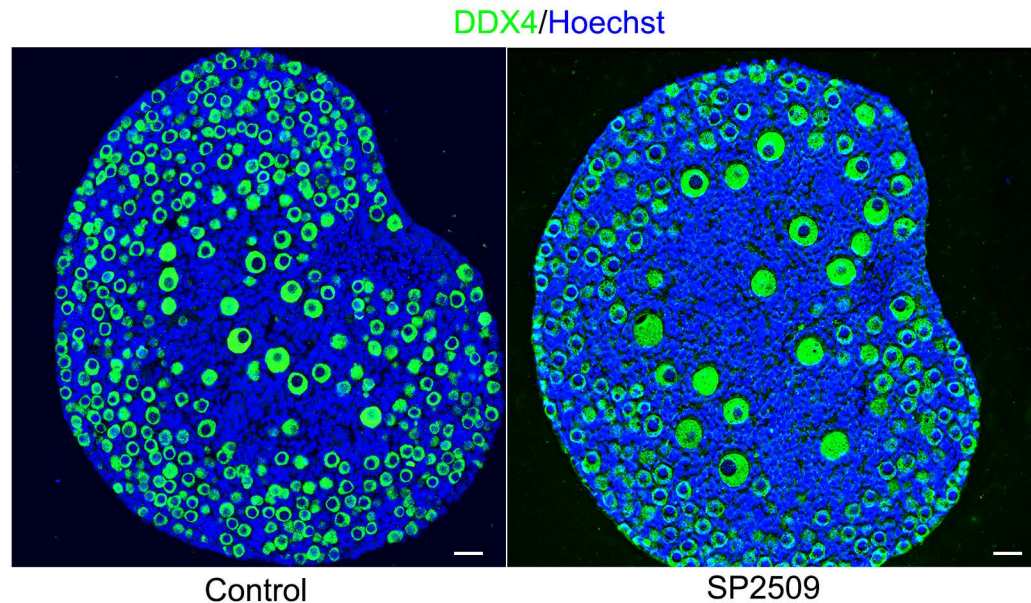

Supplement: Supplementary file 1 [file ACEL-19-e13102-s001.pdf]

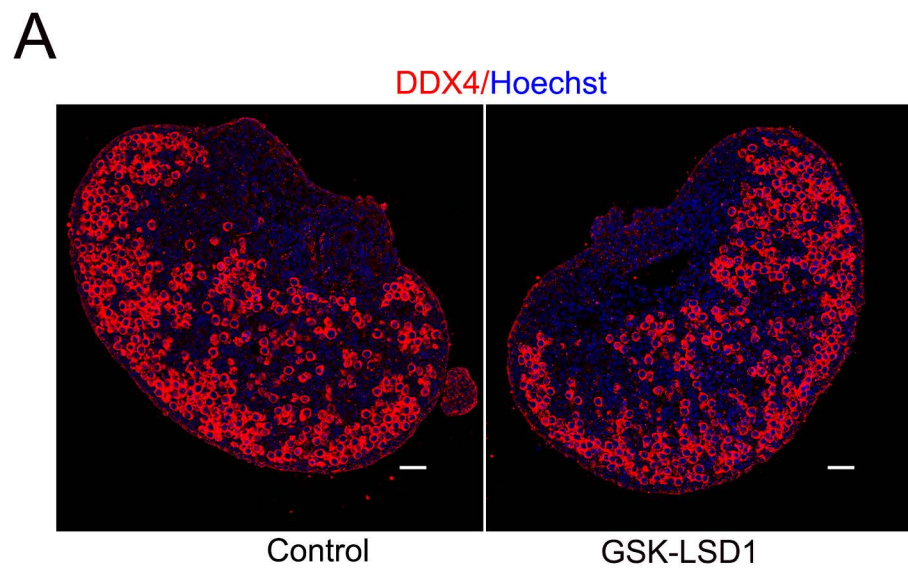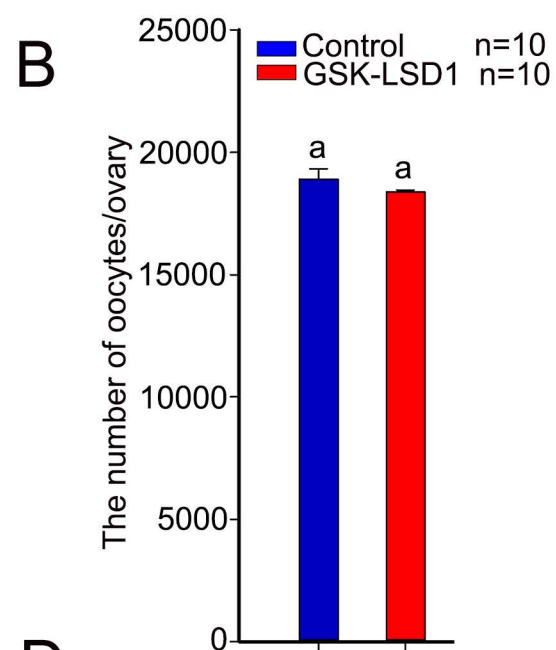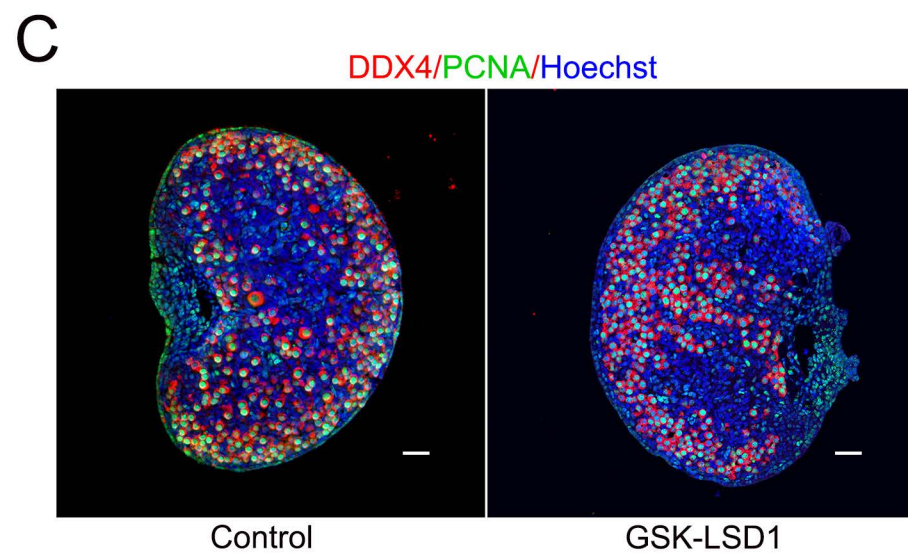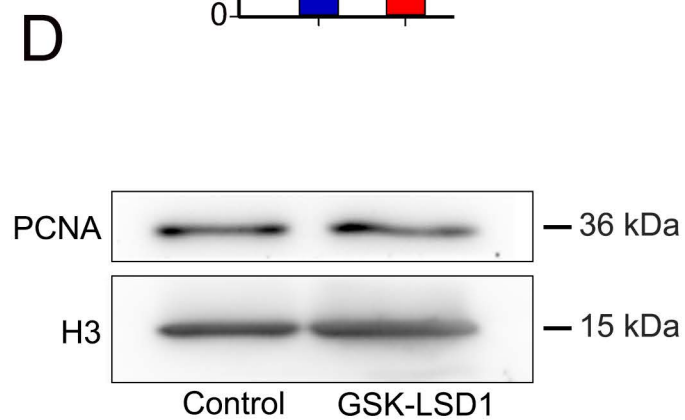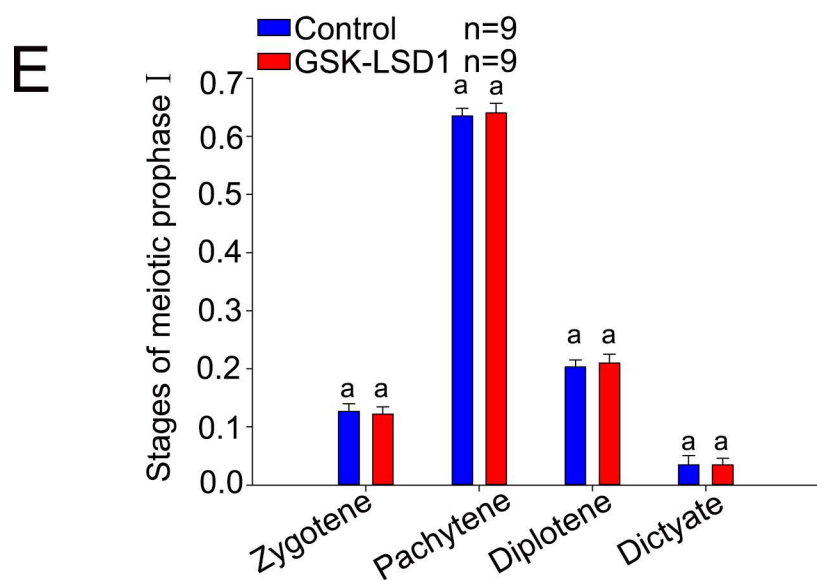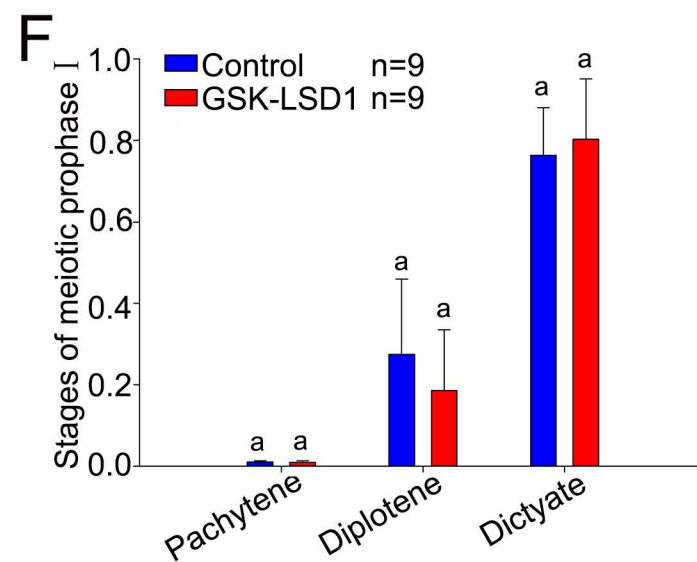

Supplement: Supplementary file 2 [file ACEL-19-e13102-s002.pdf]

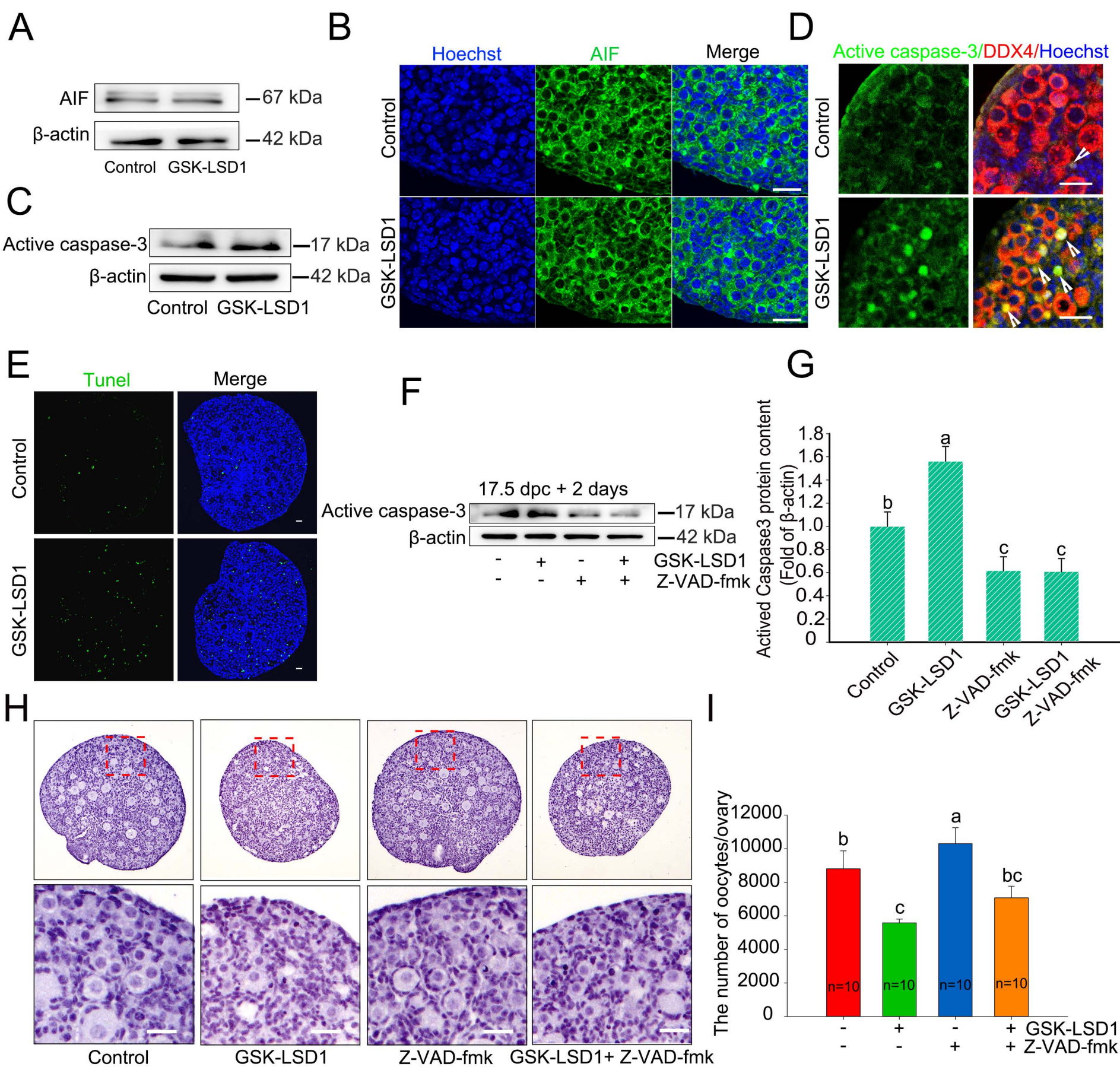

Supplement: Supplementary file 3 [file ACEL-19-e13102-s003.pdf]

17.5 dpc+2 days

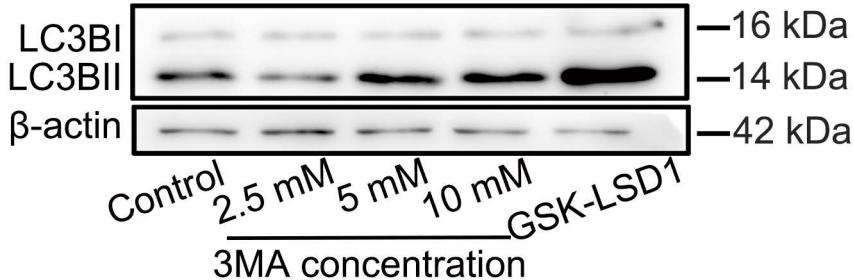

Supplement: Supplementary file 4 [file ACEL-19-e13102-s004.pdf]

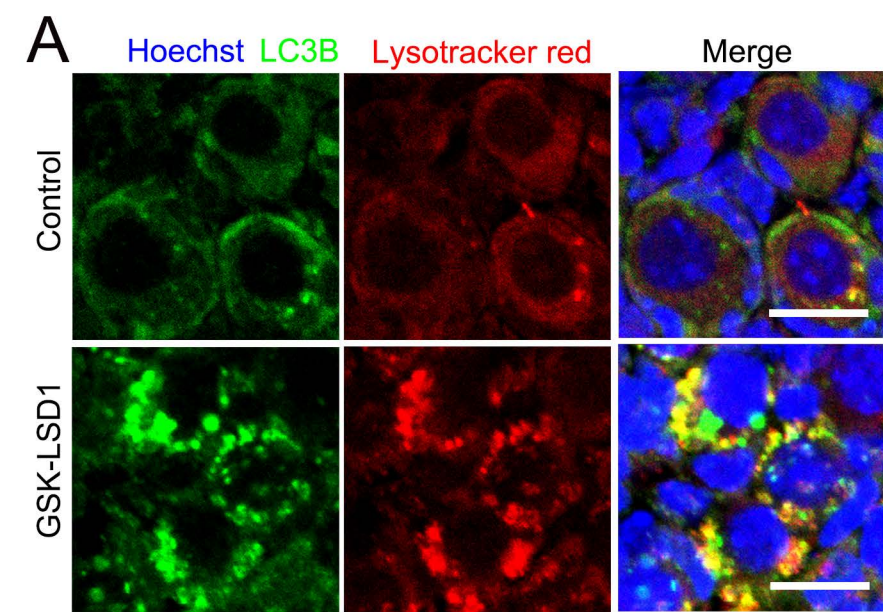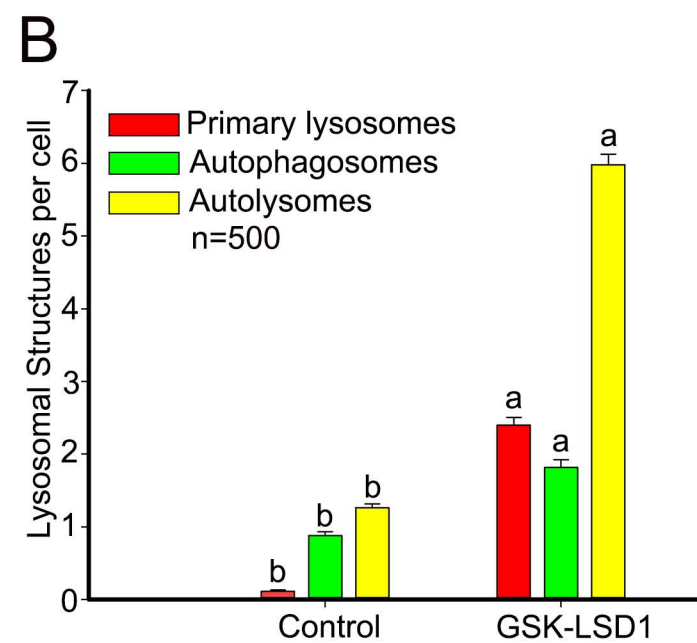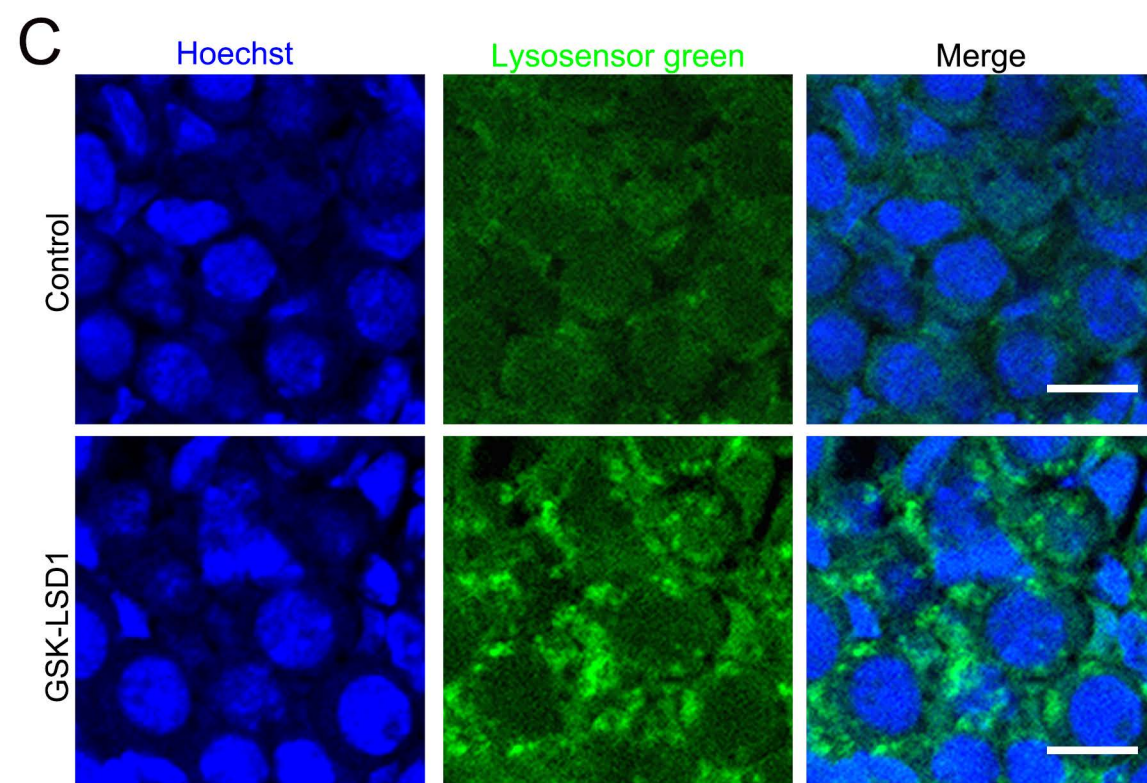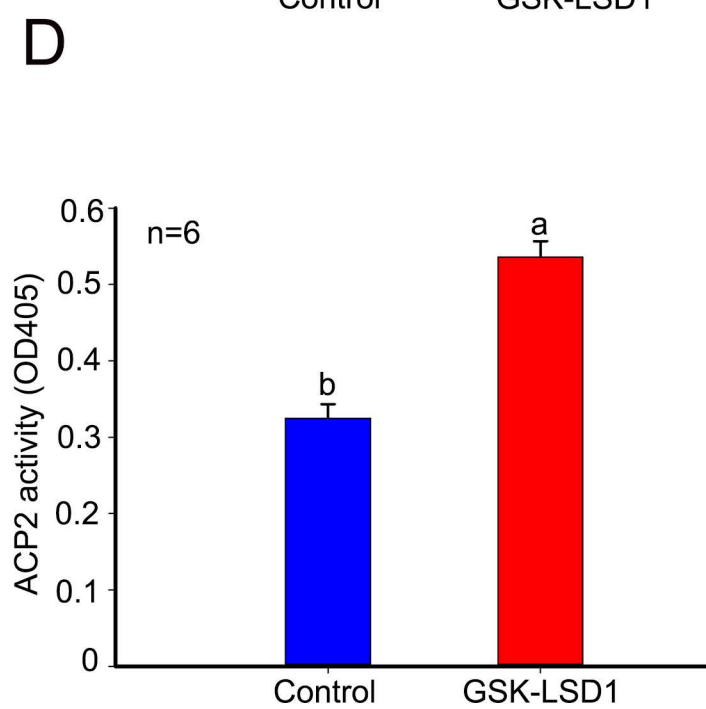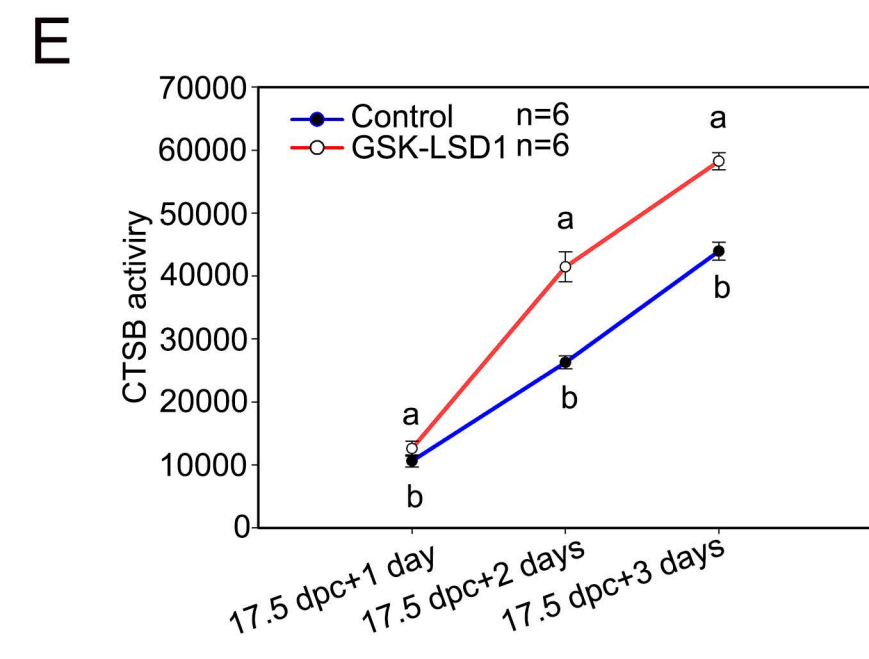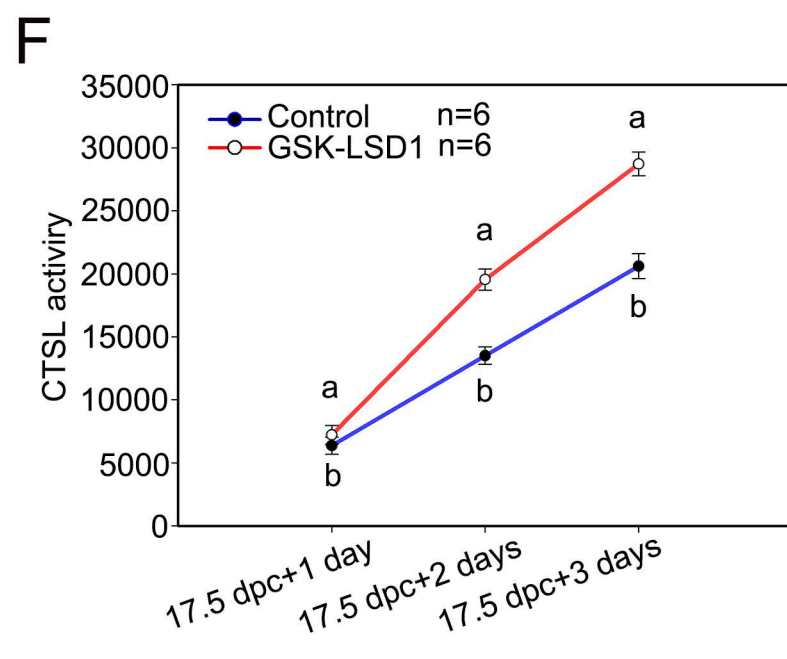

Supplement: Supplementary file 5 [file ACEL-19-e13102-s005.pdf]

**A**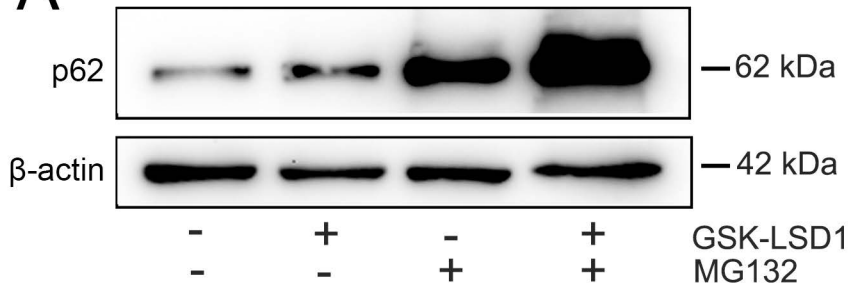**B**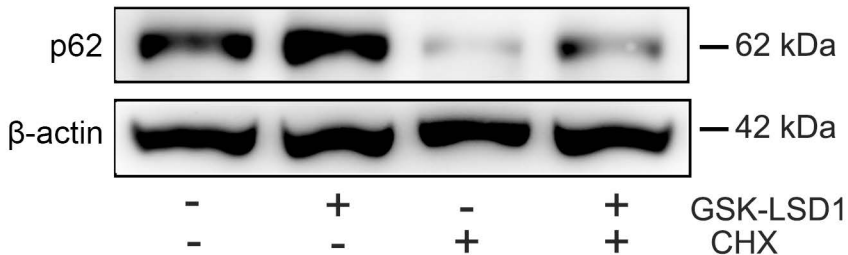

Supplement: Supplementary file 6 [file ACEL-19-e13102-s006.pdf]

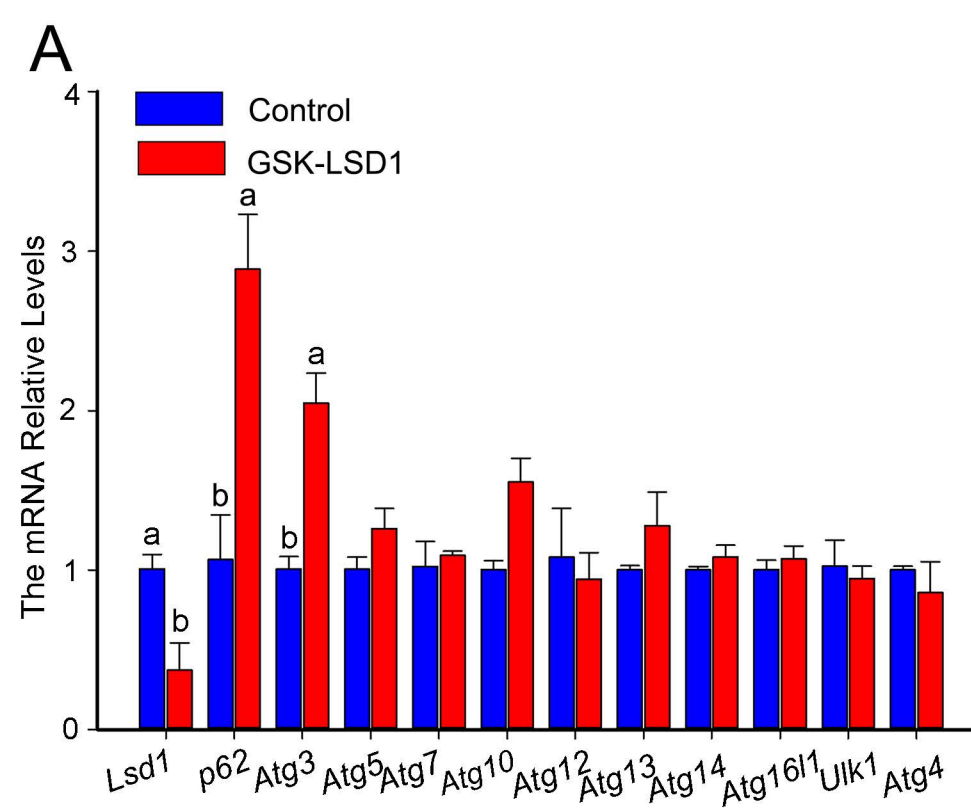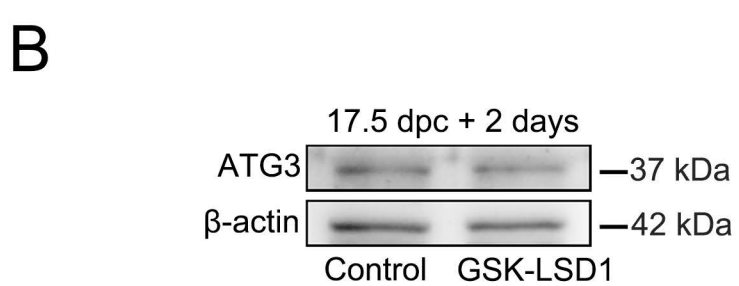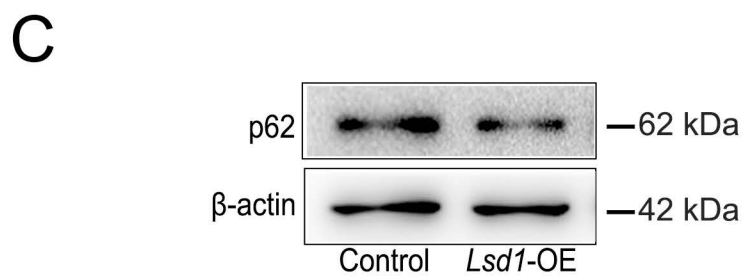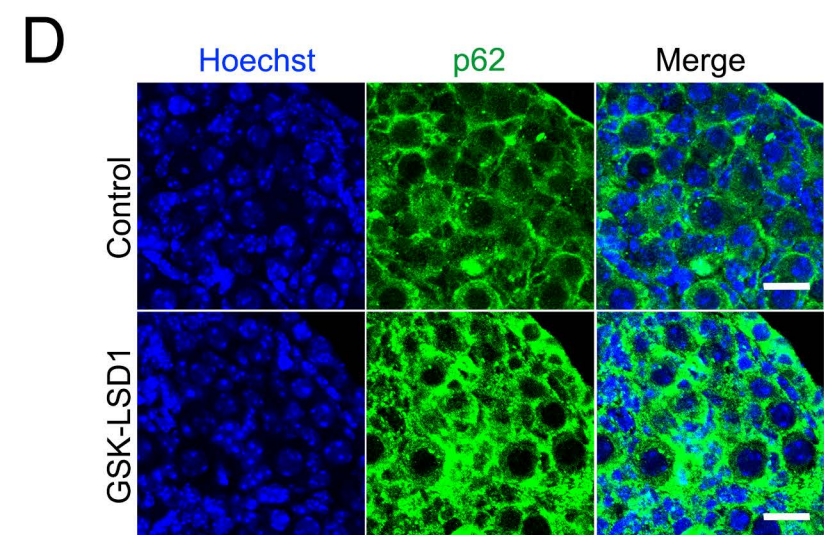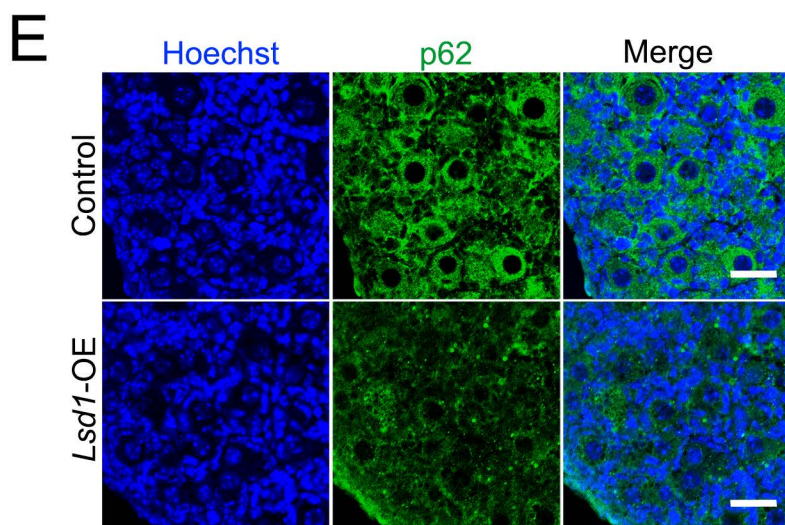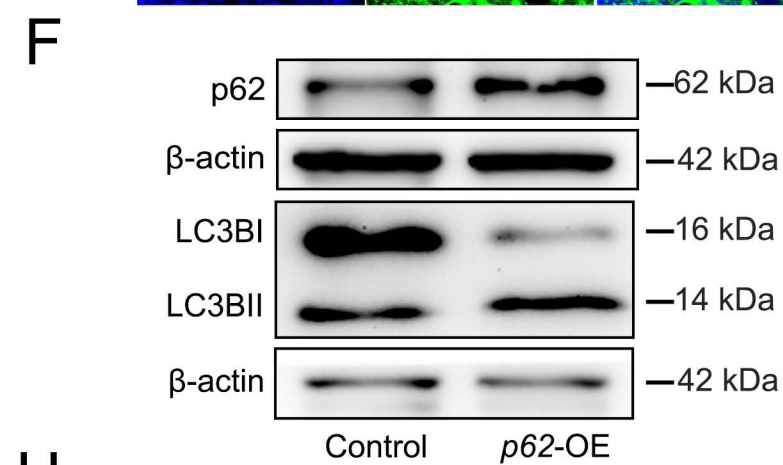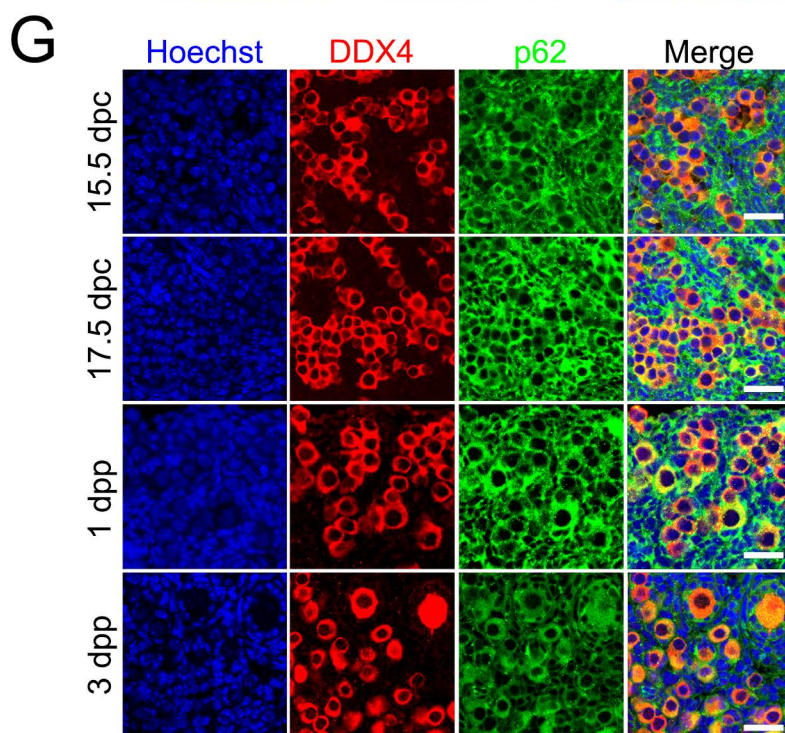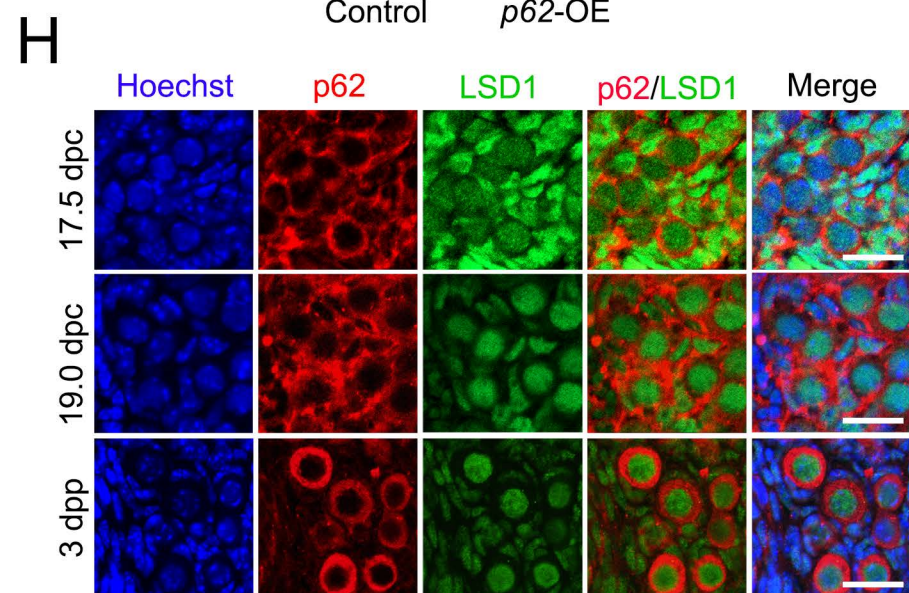

Supplement: Supplementary file 7 [file ACEL-19-e13102-s007.pdf]

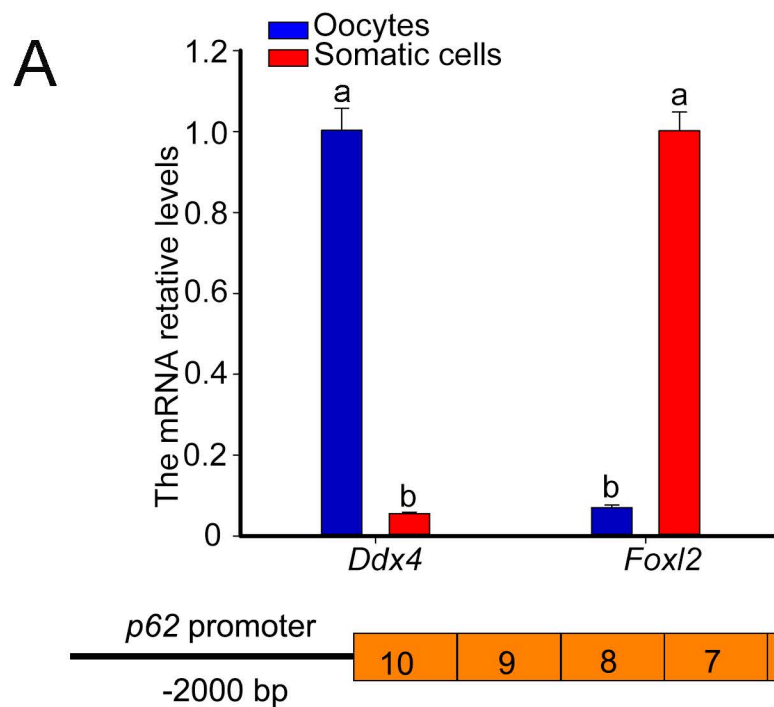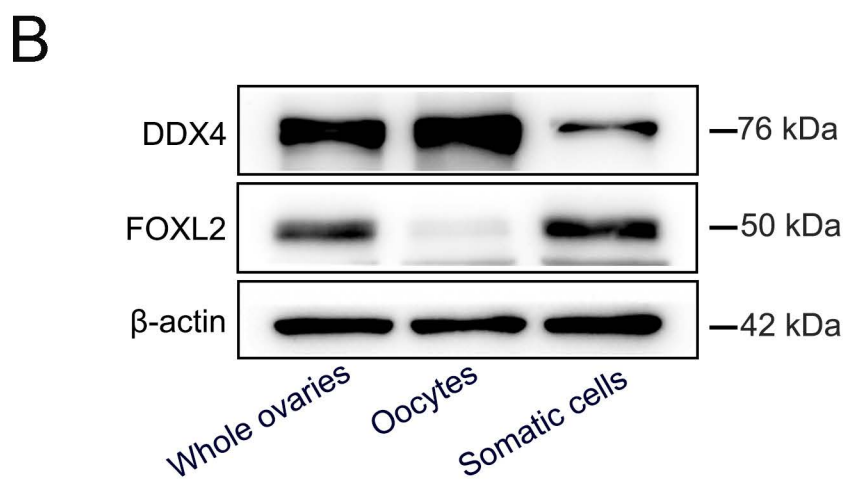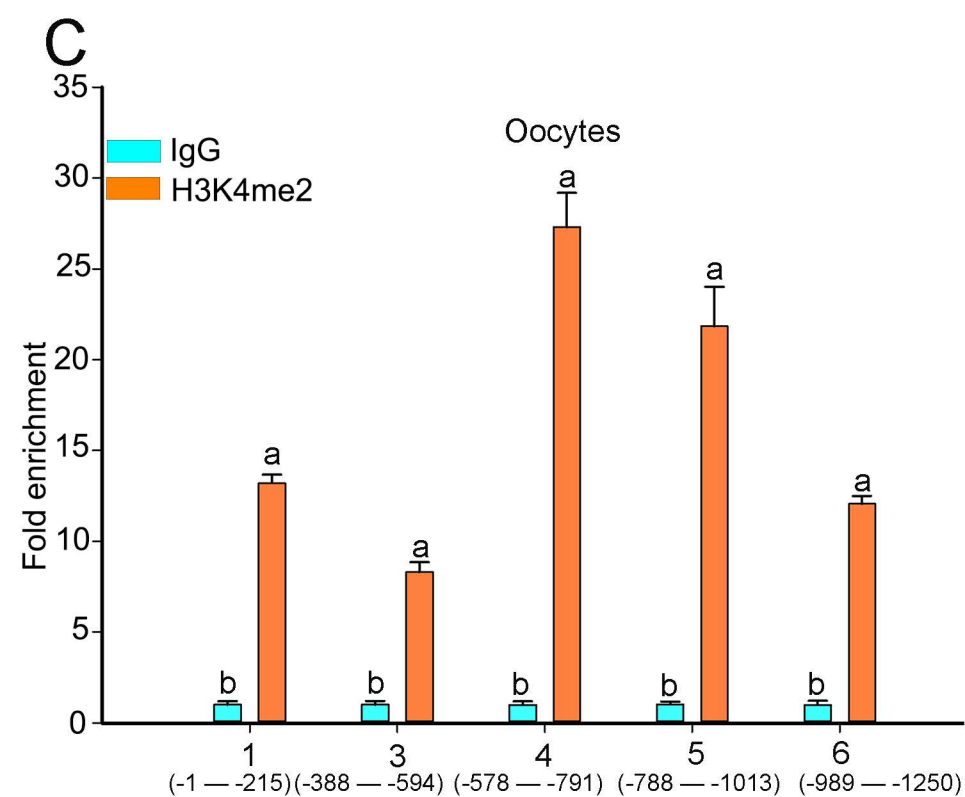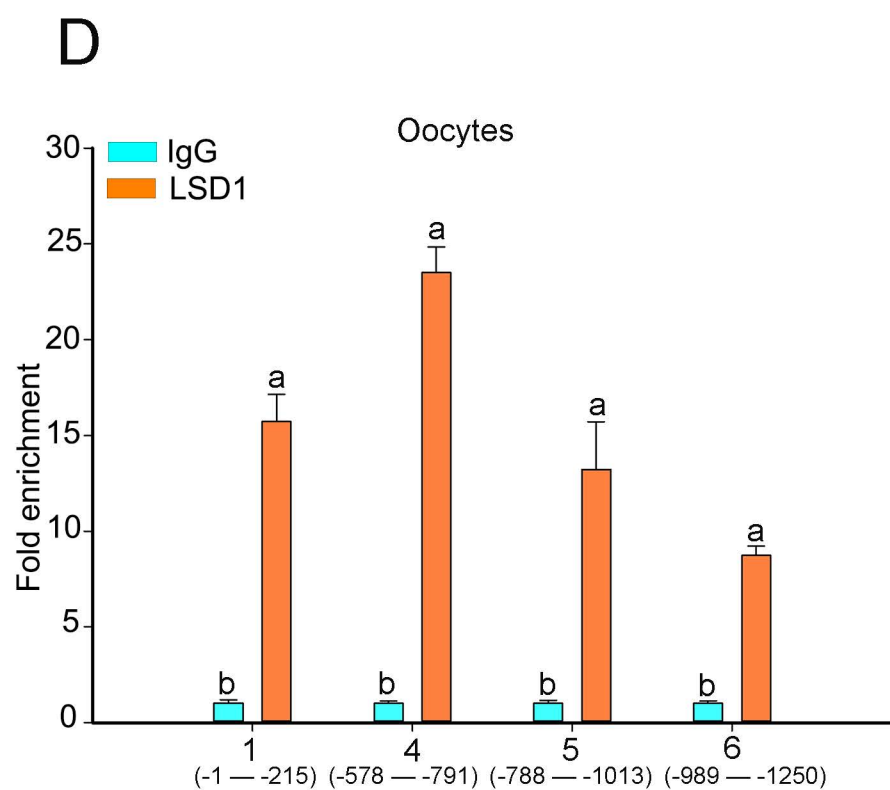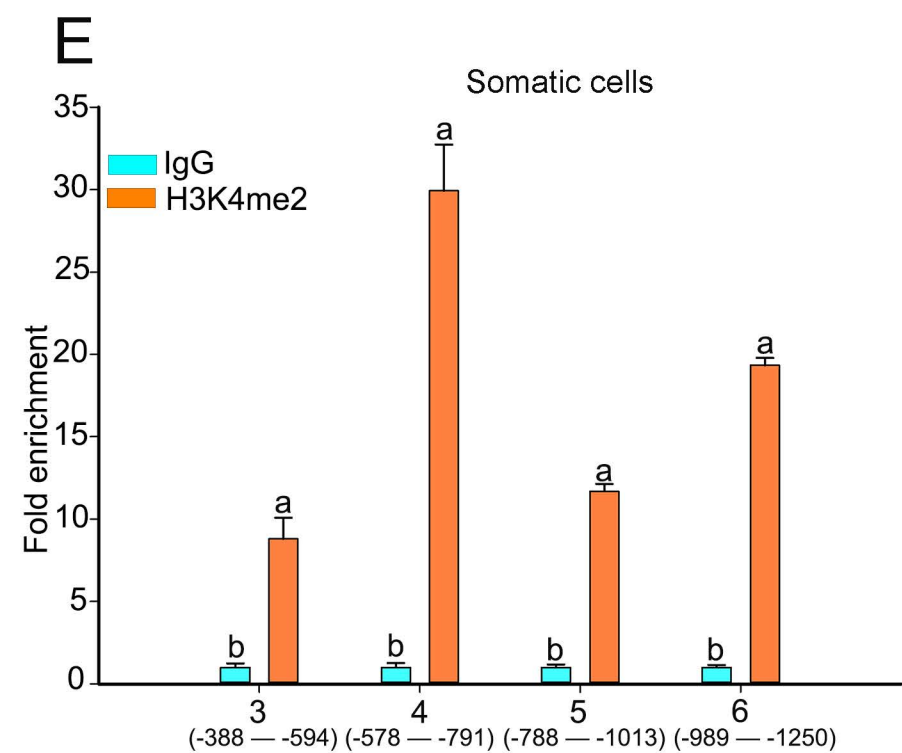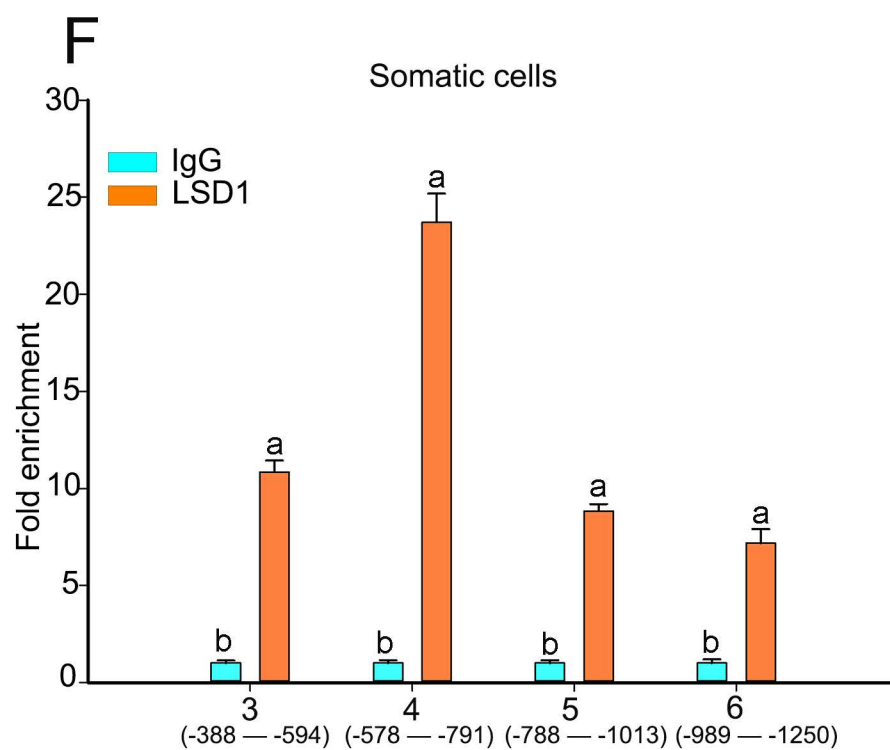

Supplement: Supplementary file 8 [file ACEL-19-e13102-s008.pdf]

Merge

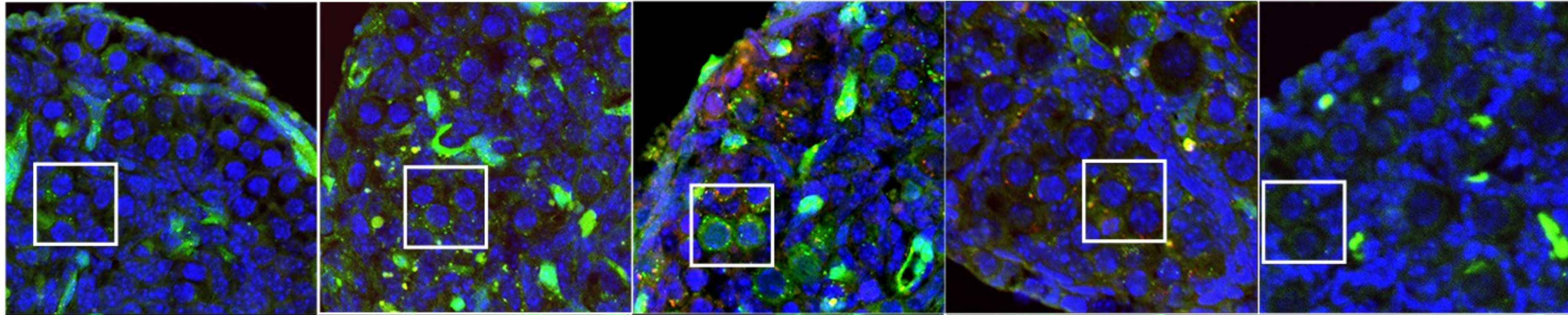

GFP-LC3

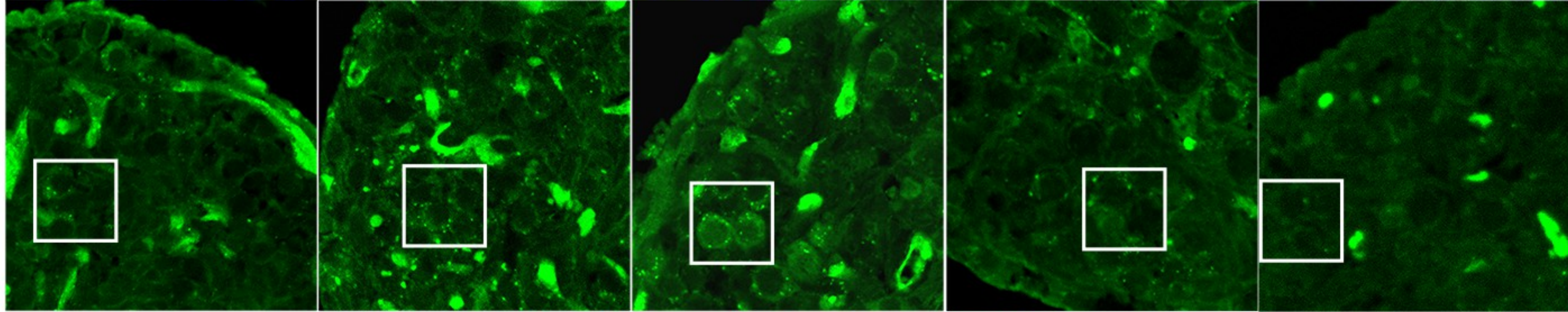

RFP-LC3

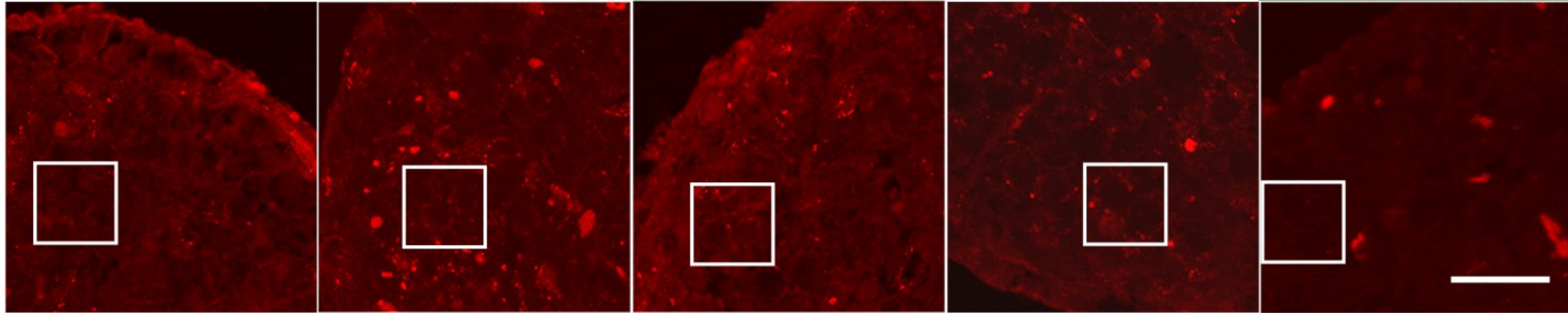

Supplement: Supplementary file 9 [file ACEL-19-e13102-s009.pdf]
