# Supplementary material for: LSD1 contributes to programmed oocyte death by regulating the transcription of autophagy adaptor SQSTM1/p62
Source: Aging Cell. 2020 Feb 19;19(3):e13102. doi: 10.1111/acel.13102 (PMC7059144; doi:10.1111/acel.13102)
Supplement: Supplementary file 10 [file ACEL-19-e13102-s010.doc]

| **List of primers** | | |
| --- | --- | --- |
| Primer | Sequence | Application |
| *Lsd1-F* | AGCGGGCCAAGGTAGAATACA | qPCR |
| *Lsd1-R* | ATGGGGAAGTCGGCTTTGAAA | qPCR |
| *β-actin-F* | GTGACGTTGACATCCGTAAAGA | qPCR |
| *β-actin-R* | GCCGGACTCATCGTACTCC | qPCR |
| *Ulk1-F* | AAGTTCGAGTTCTCTCGCAAG | qPCR |
| *Ulk1-R* | CGATGTTTTCGTGCTTTAGTTCC | qPCR |
| *Atg3-F* | GGTGATGGGGGATGGGTAGAT | qPCR |
| *Atg3-R* | AGCTTTGCAGGCTTCCACT | qPCR |
| *Atg4b-F* | GACAGGGAAGATGGACGCAG | qPCR |
| *Atg4b-R* | TAGGGCCAGTTCCCCCAATA | qPCR |
| *Atg5-F* | GACAAAGATGTGCTTCGAGATGTG | qPCR |
| *Atg5-R* | GTAGCTCAGATGCTCGCTCAG | qPCR |
| *Atg7-F* | GTTCGCCCCCTTTAATAGTGC | qPCR |
| *Atg7-R* | TGAACTCCAACGTCAAGCGG | qPCR |
| *Atg10-F* | CGAGCGAGCGGGTTCTCA | qPCR |
| *Atg10-R* | CTGGCATGTGGTGTCAAGGT | qPCR |
| *Atg12-F* | TTCCTTAAACTGGTGGCCTCG | qPCR |
| *Atg12-R* | CTCTTCCCACAGCACCGAAAT | qPCR |
| *Atg13-F* | CCAGGCTCGACTTGGAGAAAA | qPCR |
| *Atg13-R* | AGATTTCCACACACATAGATCGC | qPCR |
| *Atg14-F* | AACAACGGGGACTACTCTGC | qPCR |
| *Atg14-R* | GGTTTTCGCCACAGAACTCG | qPCR |
| *Atg16L1-F* | GATGGCTGAGAAGGCCCAAG | qPCR |
| *Atg16L1-R* | GACAGAGCGTCTCGTAGCTG | qPCR |
| *p62*-F | AGGATGGGGACTTGGTTGC | qPCR |
| *p62-*R | TCACAGATCACATTGGGGTGC | qPCR |
| *P62-p-F (KpnI)* | GGGGTACCATAAAAGCTGGGCTCTCGGCG | Promoter |
| *P62-p-R (HindIII)* | CCCAAGCTTAAGTGTATGTGTGTGCCAC | Promoter |
| *Lsd1*-OE-F (EcoRI) | CCGGAATTCATGTTGTCTGGGAAGAAGGC | Overexpression |
| *Lsd1*-OE-R (NotI) | ATTTGCGGCCGCTCACATACTTGGGGACTGCT | Overexpression |
| *p62*-OE-F (EcoRI) | GGAATTCATGGCGTCGTTCACGGTGAAG | Overexpression |
| *p62*-OE-R (BamHI) | CGGGATCCTCACAATGGTGGAGGGTGCTTCG | Overexpression |
| *p62*-OE-R (BamHI) | CGGGATCCTCACAATGGTGGAGGGTGCTTCG | Overexpression |
| *Ash1l-*i | GCCTCACAGAAAGGAACCATT | Knockdown |
| *Sqstm1/p62-i* | GAGGTTGACATTGATGTGGAA | Knockdown |
| *p62*-1-F | AGTCTCTTGCGTTGGAGTGGC | ChIP-qPCR |
| *p62*-1-R | CGGCCTAAACCACTCGTGAAT | ChIP-qPCR |
| *p62*-2-F | GCCGTCCTCAATCACTCCAATCCT | ChIP-qPCR |
| *p62*-2-R | GCACAAAGATGACTCAGAAGGAC | ChIP-qPCR |
| *p62*-3-F | TTGTCCTTCTGAGTCATCTTTGTG | ChIP-qPCR |
| *p62*-3-R | AAGGATACAAGATGACAAACAGGA | ChIP-qPCR |
| *LC3* | CATGGACGAGCTGTACAAGT | Genotyping |
| *LC3* | CACCGTGATCAGGTACAAGGA | Genotyping |
