# Supplementary material for: LSD1 contributes to programmed oocyte death by regulating the transcription of autophagy adaptor SQSTM1/p62
Source: Aging Cell. 2020 Feb 19;19(3):e13102. doi: 10.1111/acel.13102 (PMC7059144; doi:10.1111/acel.13102)
Supplement: Supplementary file 11 [file ACEL-19-e13102-s011.doc]

| Antibody | Host | WB | IF/IHC | Supplier |
| --- | --- | --- | --- | --- |
| LSD1 | Rabbit | 1:500 | 1:100 | Cell Signaling Technology, 2139 |
| H3K4me2 | Rabbit | 1:500 | 1:100 | Abcam,ab 32356 |
| H3K4me | Rabbit | 1:500 |  | Abcam, ab8895 |
| H3K9me2 | Rabbit | 1:500 |  | Abcam, ab176882 |
| H3K9me | Rabbit | 1:500 |  | Abcam, ab9045 |
| ASH1L | Rabbit | 1:250 | 1:100 | Bioss, 11866R |
| LC3B | Rabbit | 1:500 | 1:100 | Cell Signaling Technology, 2775 |
| p62 | Mouse | 1:500 | 1:100 | Abcam, ab56416 |
| ATG3 | Rabbit | 1:500 |  | Bioworld, BS7872 |
| mTOR | Rabbit | 1:500 |  | Cell Signaling Technology, 2983 |
| Caspase-3 | Rabbit | 1:500 |  | Cell Signaling Technology, 9662 |
| Active caspase-3 | Rabbit |  | 1:100 | Beyotime Biotechnology, AC033 |
| AIF | Rabbit | 1:500 | 1:100 | Beyotime Biotechnology, AA306 |
| DDX4 | Rabbit |  | 1:300 | Abcam, ab13840 |
| DDX4 | Mouse |  | 1:300 | Abcam, ab27591 |
| β-actin | Mouse | 1:1000 |  | CWbiotech, CW0098M |
| SYCP3 | Rabbit |  | 1:100 | Novus Biologicals, NB300-232 |
| H3 | Rabbit | 1:1000 |  | Abcam, ab1791 |
| H3K36me | Rabbit | 1:1000 |  | Abcam, ab9048 |
| PCNA | Mouse | 1:1000 | 1:100 | Santa Cruz, sc-56 |
| H3K36me2 | Rabbit | 1:1000 |  | Abcam, ab9049 |
| H3K4me3 | Rabbit | 1:1000 |  | Cell Signaling Technology,9751 |

**List of antibodies**
