# Supplementary material for: LSD1 contributes to programmed oocyte death by regulating the transcription of autophagy adaptor SQSTM1/p62
Source: Aging Cell. 2020 Feb 19;19(3):e13102. doi: 10.1111/acel.13102 (PMC7059144; doi:10.1111/acel.13102)
Supplement: Supplementary file 12 [file ACEL-19-e13102-s012.doc]

**Figure legends**

**Fig. S1** **Inhibition of LSD1 by SP2509 leads to both the decrease of LSD1 protein level and oocytes loss in cultured ovaries.**

**(A)** Inhibition of LSD1 by SP2509 significantly decreased the level of LSD1 in cultured ovaries. **(B-C)** SP2509 significantly reduced the oocytes number in cultured ovaries.

**Fig. S2 Effect of LSD1 is time-dependent.**

**(A, B)** Morphological analysis and counting result showed that GSK-LSD1 treated ovaries had no differences than the control. 13.5 dpc ovaries were cultured with GSK-LSD1 for 4 days. **(C, D)** PCNA protein expression was not obviously different after treatment with GSK-LSD1 for 4 days. PCNA: green; the oocyte marker DDX4: red; nuclei: blue. **(E, F)** Ovaries were cultured from 15.5 dpc to 18.0 dpc and from 17.5 dpc to 1 dpp by chromosome spread. Each period of meiosis I was not different compared to that of the control. The different letters above the bars indicate significant differences. Scale bars, 25 μm.

**Fig. S3 Apoptosis is involved in the massive oocyte loss, but is not the major cause.**

**(A, B)** The protein level and cytoplasm localization of AIF were unchanged after 17.5 dpc ovaries were cultured with GSK-LSD1 for 2 days. AIF: green; nuclei: blue. **(C-E)** Evidence of apoptosis after 17.5 dpc ovaries were cultured with GSK-LSD1 for 2 days. **(C)** The expression of active caspase-3 was elevated significantly. **(D)** Active caspase-3 was evidenced in nuclei of oocytes (arrows). Active caspase-3: green; the oocyte marker DDX4: red; nuclei: blue. **(E)** Fetal mouse ovaries from 17.5 dpc were cultured *in vitro* with DMSO or GSK-LSD1 for 2 days before apoptosis was evaluated. TUNEL signal: green; nuclei: blue. **(F-I)** GSK-LSD1 counteracted to the effect of Z-VAD-fmk on stimulating oocyte apoptosis, as indicated by the level of active caspase-3. **(F-G)** Inhibiting LSD1 and apoptosis can efficiently inhibit the expression of active caspase-3. **(H-I)** Morphological analysis and statistical evidence. 17.5 dpc ovaries were cultured with chemicals for 2 days or 6 days. The different letters above the bars indicate significant differences. Scale bars, 25 μm.

**Fig.S4 An assay to determine the optimized concentration of 3MA.**

Two days of 3MA treatment (2.5 mM) inhibited autophagy.

**Fig. S5 LSD1 inhibition does not block the degradation function of lysosomal.**

1. Autophagosomes and lysosomes can fuse together. Lysosomes was probed with LysoTracker Red DND-99. Scale bars: 10 μm. LC3B: green; LysoTracker Red: red; nuclei: blue. **(B)** The counting results of Lysosomal structure per cell. **(C)** The acidification ability of lysosomes was measured by Lysosensor™ Green. Scale bars: 10 μm. Lysosensor™ Green: green; nuclei: blue. **(D)** An *in vitro* ACP2/acid phosphatase activity assay was performed after 3 days of GSK-LSD1 treatment. **(E-F)** The quantification of lysosomal CTSB/CTSL activity 1, 2, and 3 days after GSK-LSD1 treatment. CTSB/CTSL activity was visualized with a Magic Red Cathepsin B probe.The different letters above the bars indicate significant differences. Scale bars, 25 μm.

**Fig. S6 LSD1 inhibition does not affect The ubiquitin-proteasome system (UPS) activity.**

**(A)** The protein level of p62 was significant increased in GSK-LSD1 plus MG132 treatment compared with GSK-LSD1 or MG132 treatment. **(B)** The protein level of p62 was significant decreased in GSK-LSD1 plus CHX treatment compared with GSK-LSD1 or Control treatment. 17.5 dpc ovaries were cultured with or without GSK-LSD1 for 1 day, and then MG132 or CHX were added and cultured for another 1 day.

**Fig. S7** **H3K4me2 regulates *p62* transcription.**

**(A)** Changes in the mRNA levels of *Ulk1*, *p62* and ATGs after 17.5 dpc ovaries were cultured with GSK-LSD1 for 2 days. **(B)** The protein level of ATG3 did not change after the ovaries were cultured with GSK-LSD1. **(C)** The p62 protein level was decreased after *Lsd1* was overexpressed. **(D-E)** The p62 protein levels increased or decreased correspondingly after LSD1 was inhibited **(D)** or overexpressed **(E)**, correspondingly. p62: green; nuclei: blue. **(F)** p62 expression and LC3 conversion were obviously upregulated in *p62*-OE ovaries after 3 days of culture. LSD1 and Ash1L expression didn’t change significantly. **(G-H)** The cellular localization of p62 in fetal ovaries. P62 is located in the cytoplasm of both oocytes and somatic cells. **(G)** p62: green; the oocyte marker DDX4: red; nuclei: blue. **(H)** p62: red; LSD1: green; nuclei: blue. The different letters above the bars indicate significant differences. Scale bars, 25 μm.

**Fig. S8 ChIP-qPCR showed that both LSD1 and H3K4me2 directly occupy respective regions of the *p62* promoter. This binding phenomenon occurs in both oocytes and somatic cells.**

**(A, B)** The evaluation of the separation efficiency of somatic cells (indicated by FOXL2) and oocytes (indicated by DDX4) by real-time qPCR **(A)** and Western blot **(B)**. **(C)** H3K4me2 directly occupies the -1250 bp to -1 bp region of the *p62* promoter in oocytes. **(D)** LSD1 directly occupies the -1250 bp to -578 bp region and the -215 bp to -1 bp region of the *p62* promoter in oocytes. **(E)** H3K4me2 directly occupies the -1250 bp to -388 bp region of the *p62* promoter in somatic cells. **(F)** LSD1 directly occupies the -1250 bp to -388 bp region of the *p62* promoter in somatic cells. The different letters above the bars indicate significant differences.

**Fig. S9 Morphological analysis of autophagic activity under physiological conditions.**

Ovaries were obtained from LC3-GFP-RFP mice 17.5 dpc, 18.5 dpc, 1 dpp, 2 dpp, and 3 dpp. Amplified views of the boxed areas are shown in Fig. 6A. Scale bars, 25 μm.
